# Carnosine Biofunctionalized Hydroxyapatite Induces Copper-Driven Osteogenesis and Angiogenesis, Strengthening Its Bone Regenerative Capacities

**DOI:** 10.1021/acsbiomaterials.5c00823

**Published:** 2025-08-31

**Authors:** Irina Naletova, Francesco Attanasio, Teresa Sibillano, Barbara Tomasello, Valeria Lanza, Valeria Ciaffaglione, Rita Tosto, Antonio Mio, Warren Cairns, Cinzia Giannini, Enrico Rizzarelli

**Affiliations:** 1 567341 Institute of Crystallography, National Council of Research, CNR-IC, Via P. Gaifami 18, Catania 95126, Italy; 2 Institute of Crystallography, National Council of Research, CNR-IC, Via G. Amendola 122, Bari 70126, Italy; 3 Department of Drug and Health Sciences, 9298 University of Catania, V.le A. Doria 6, Catania 95125, Italy; 4 Institute for Microelectronics and Microsystems, National Council of Research, CNR-IMM, Zona Industriale Strada VIII n. 5, Catania 95121, Italy; 5 Institute of Polar Sciences, National Council of Research, CNR-ISP, c/o Campus Scientifico, Università Ca’ Foscari Venezia Via Torino, 155, Venezia Mestre 30170, Italy; 6 Department of Chemical Sciences, 9298 University of Catania, V.le A. Doria 6, Catania 95125, Italy

**Keywords:** hydroxyapatite, carnosine, copper homeostasis, osteogenesis, growth factors

## Abstract

Hydroxyapatite (Hap) is a prominent biomaterial used as an effective implant material in bone tissue engineering, but its use presents some points of weakness in bone regeneration efficiency. Different biofunctionalization strategies have been utilized to increase the regenerative Hap capacities. Carnosine (Car) or β-alanyl-l-histidine dipeptide has received much attention due to its beneficial effects in osteoarticular diseases and bone tissue healing. Hap functionalized in noncovalent mode with Car at a nominal Ca:Car molar ratio (10:1, 2:1, and 1:1) was synthesized. The Hap-Car composites were characterized by using X-ray diffraction, scanning electron microscopy, and Fourier transform infrared spectroscopies. The structural and morphological feature comparisons indicate a similarity between Hap-Car10:1 and Hap. The Hap-Car composites and Hap bind copper present at submicromolar concentration in the complete culture medium, determined by inductively coupled plasma-optical emission spectroscopy. Hap-Car composites enhance the biological properties of Hap in in vitro assays and promote the mineralization process and the expression of alkaline phosphatase, osteocalcin, vascular endothelial growth factor, brain derived neurotrophic factor, and bone morphogenetic protein-2 in hFOB1.19 cells. The protective and regenerative activities of the metal ion are also related to the intracellular chaperone copper chaperones for superoxide dismutase.

## Graphical abstract

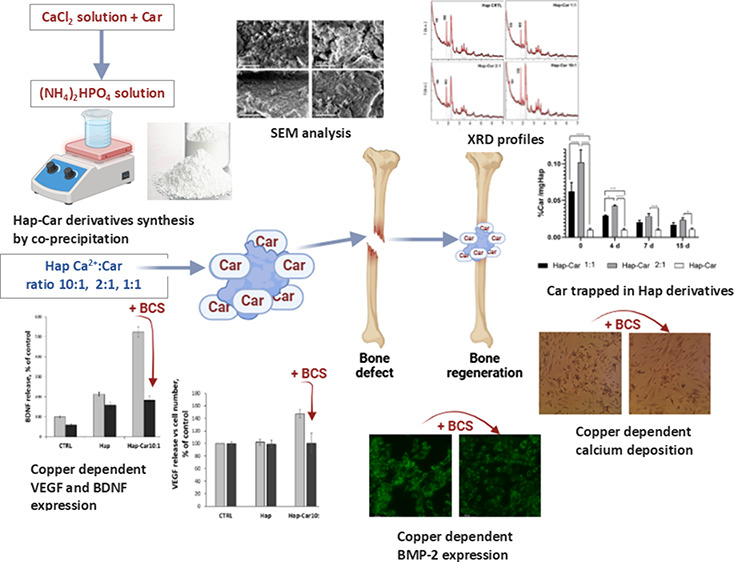

## 1 Introduction

Bone tissues contain both organic and inorganic components in addition to approximately 8–10% of water. Inorganic species account for 60% of the mass, while the remaining percentage is the organic component.^1^ Type I collagen and different noncollagenous proteins are the major organic species, together with cells (approximately 2%) that are necessary for bone function. The main mineral components are calcium salts, which include calcium hydroxyapatite (Hap), calcium carbonate, and magnesium phosphate. Bone undergoes continuous remodeling^2^ that requires the coordinated activities of three main types of cells: osteoblasts, osteoclasts, and osteocytes.^3^ The orchestrated activities of these cell types regulate skeletal development, remodeling, and regeneration.^4^

Bone is a highly vascularized tissue^5^ where blood vessels provide the skeletal system with oxygen, nutrients, specific hormones, growth factors, and neurotransmitters, supporting bone development, regeneration, and remodeling,^6–8^ by utilizing the tight link between blood vessel growth (angiogenesis) and the formation of new bone (osteogenesis).^9–11^ In this context, the secretion of vascular endothelial growth factor (VEGF) is essential for coupling osteogenesis with angiogenesis.^5^

Despite these features, the bone’s ability to self-repair its damaged tissues is principally restricted to small parts of bone tissue; thus, bone tissue engineering^12^ is an alternative solution to assist self-repair and restore large defects. A restricted number of bone engineering scaffolds meet all the essential requirements of an attractive scaffold^13,14^ such as biocompatibility, bioactivity, porosity, mechanical properties, biodegradability, and structural stability.^15^ To satisfy these criteria, and due to their composition, that matches the inorganic components of bone, bioceramics have captured significant interest in the field of bone tissue engineering.^16^ Their high biocompatibility, bioactivity, chemical stability, and suitable mechanical and physical features give reason for their prominence^17,18^ in regulating bone regeneration.^19–21^

Natural hydroxyapatite (nat-Hap) is the major inorganic species in bone tissue, as it provides a store of calcium and phosphorus, significantly contributing to bone healing.^22^ Recently, the potential exposure to disease transmission when employing bovine-derived bone substitutes has become more evident, underlining the need for a synthetic graft material with similar bioactive features.^23^ Therefore, synthesized Hap with the chemical formula Ca_10_(PO_4_)_6_(OH)_2_ is the major inorganic substitute for bone because of its chemical and crystallographic structure that is similar and isomorphic to that of nat-Hap. Hap shows stable physical properties, significant biocompatibility, and osteoinductive capacity.^24,25^ In recent years, Hap-based scaffolds have emerged as promising biomaterials for the development of bone regenerative agents; they promote remineralization, osteoblast survival, adhesion, and proliferation, as well as increasing alkaline phosphatase activity, osteogenic differentiation, and bone-specific gene expression.^4^ Lately, hydroxyapatite nanoparticles (n-Hap) have been also employed in different biomedical fields,^26^ leveraging their (i) high surface-to-volume ratio, (ii) high surface reactivity, (iii) high binding affinities for proteins and nucleic acids, (iv) internalization ability in diverse cell types, (v) faster biodegradation in comparison to other inorganic materials, and (vi) pH-dependent solubility.^27,28^

Thus, n-Hap has been intensively investigated to promote (intracellular) drug delivery in which efficient cellular uptake and drug release are required.^28,29^ Although hydroxyapatite is an attractive scaffold, it presents several diverse disadvantages, which are (i) low body absorption, (ii) low mechanical strength, and (iii) limited bone regeneration efficiency compared to autologous bone due to its insufficient angiogenetic and osteogenic abilities.^30–32^ With the aim of maximizing bone regenerative efficiency, organic/hydroxyapatite, polymer/hydroxyapatite, and metal ion-doped/hydroxyapatite composites are continuously being investigated.^33–37^

To build a favorable vascularized and osteogenic microenvironment for bone regeneration, growth factors including VEGF and BMP-2 (bone morphogenetic protein-2) are employed to enhance the angiogenic and osteogenic ability of synthetic hydroxyapatites.^38–41^ Although different biofunctionalization strategies are described,^16^ their high manufacturing cost, negative effects on protein stability, and the development of unwanted immunogenic responses, together with the risk of adverse effects due to supraphysiological doses of the growth and trophic factors, prevent their widespread usage. Osteoinductive naturally derived small molecules^42^ can represent an alternative to proteins; these low molecular weight compounds can diffuse across the cellular membranes to activate signaling cascade pathways toward osteogenic differentiation^43,44^ and angiogenesis.^45^ Recently, enhanced bone regenerative properties of hydroxyapatite have been obtained by its noncovalent functionalization with a VEGF mimetic peptide,^46^ QK,^47^ or BMP-2 mimic peptide,^31^ KP.^48^ Lately, the entrapment of both KP and QK in an injectable and self-healing hydrogel displayed synergistic osteogenic and angiogenic effects, promoting efficient bone regeneration.^49^

Carnosine (β-alanyl-l-histidine, Car) is a natural dipeptide that is produced by the cytosolic enzyme, carnosine synthetase,^50^ mainly in skeletal muscle^51,52^ and the olfactory bulb, where its concentration is in the mM range.^53^ Car shows pH buffering activity, chelates transition metals, scavenges lipid peroxidation products,^54^ and displays protective effects against damages caused by reactive species derived from oxygen (ROS), nitrogen (RNS), or carbonyl species (RCS) due to its scavenging capacities.^40,55,56^ Car displays anti-inflammatory, antiaging, antiaggregating, anticrosslinking, antitumor, and positive immune regulatory activities in vitro and in vivo.^57–66^ However, the therapeutic uses of carnosine are limited by its low bioavailability, because the dipeptide is rapidly degraded by serum^67,68^ and tissue^69^ carnosinase, which hydrolyze carnosine into β-alanine and l-histidine. Therefore, carnosine derivatives have been synthesized using different approaches,^70–75^ including dipeptide conjugation with polysaccharides to inhibit or delay Car hydrolysis.^76–80^

Recent studies provide evidence for the protective effects of Car in bone and cartilage injuries thanks to its antioxidant activity; Car also promotes bone formation, giving rise to osteoblasts’ proliferation and activity, impeding osteoclasts’ bone resorption, and tuning the differentiation of bone marrow MSCs (mesenchymal stem cells).^81^ The dipeptide may also improve angiogenesis^82^ and osteogenesis.^83^ More recently, the beneficial effects of Car in osteoarticular diseases and bone tissue regeneration have also been ascribed to the copper and zinc chelating ability of the dipeptide,^84–86^ highlighting the increasing appreciation of the Cu^2+^ role in restoration of bone and joint defects at a physiological range of concentrations.^87–92^

As we are aware that bone tissue regeneration is accomplished by means of the concerted effects of osteogenesis and angiogenesis, we have attempted to build a favorable regenerative microenvironment by incorporating the dipeptide into a synthetic hydroxyapatite, obtaining the new organic/hydroxyapatite composite Hap-Car, to promote both the vascularization and osteogenesis capabilities of the scaffold. For the purpose of our study, we evaluated some chemical and biological properties of this composite at different loadings of the dipeptide covering the stoichiometric ratios of Hap-Car10:1, Hap-Car2:1, and Hap-Car1:1. The physical and chemical features of the Hap-Car scaffolds were studied by different techniques: X-ray diffraction (XRD), scanning electron microscopy (SEM), and Raman and Fourier transform infrared (FT-IR) spectroscopies were performed, while their biological activities were tested in the immortalized human fetal osteoblast cell line hFOB1.19.

These new Hap-based biomaterials doped with Car show good biocompatibility, induce morphological changes, promote osteoblast differentiation, and activate the osteogenic markers BMP-2, osteocalcin (OSC), alkaline phosphatase (ALP), and biomineralization. In addition, the VEGF release is significantly raised by Hap-Car composites as well as the trophic protein brain derived neurotrophic factor (BDNF), which can promote hBMSC osteogenesis, both *in vitro* and *in vivo*.^93^

These Hap-Car derivatives affect the above-mentioned processes more than Hap, increasing the potential of its osteogenic and bone regeneration features. Moreover, all the capacities of Hap and the Hap-Car derivatives are increased by the contribution of copper that is present in the complete culture medium at physiological concentrations, the levels of which were determined by inductively coupled plasma-optical emission spectroscopy (ICP-OES). Furthermore, Hap-Car scaffolds and, to a lesser extent, Hap affect the levels of the membrane copper transporter 1 (Ctr1), the copper intracellular chaperone, copper chaperone for superoxide dismutase (CCS), and P-type copper-transporting ATPase (ATP7B)^94^ involved in copper homeostasis and metal ion signaling pathways.^95^

## 2 Materials and Methods

Details on the materials and methods used are provided in the Supporting Information.

### 2.1 Reagents

All reagents were provided by Sigma-Aldrich unless otherwise specified. All solutions used in experiments were prepared in Milli-Q water.

### 2.2 Synthesis of Hap-Car Derivatives

Hap-Car derivatives were synthesized by coprecipitation of calcium, Car, and phosphate solutions.

### 2.3 X-ray Diffraction (XRD)

XRD profiles were collected using a diffractometer equipped with an 18 kW rotating anode (with a copper target), an asymmetric Johansson Ge(111) crystal to isolate monochromatic Cu Kα_1_ radiation (λ = 1.54056 Å), a horizontal 2θ/θ goniometer, and a silicon strip D/teX Ultra detector.

### 2.4 Scanning Electron Microscopy (SEM)

The surface morphology of the powders was investigated with a field emission scanning electron microscope (FESEM) using a Thermo Scientific Helios 5 UC Dual Beam system equipped with a through lens detector (TLD), polarized to acquire secondary electrons for high resolution imaging in immersion mode. The powders were sonicated for 15 min and dispersed on a copper tape, and the excess was removed.

### 2.5 Fourier-Transform Infrared (FT-IR) Spectral Analysis

FT-IR spectroscopy was used to define the functional groups of the Hap and Hap-Car derivatives. The spectra were acquired using a Thermo Scientific Nicolet iS10 FT-IR Spectrometer.

### 2.6 Carnosine Loading

Peptide loading on the Hap-Car derivatives was determined using a spectrofluorimetric method, as reported previously^96^ and detailed in the Supporting Information.

### 2.7 Inductively Coupled Plasma-Optical Emission Spectroscopy and Mass Spectrometry

The concentration of copper was quantified using a Thermo Scientific iCAP 7400 Duo ICP-OES. The concentrations, especially the lower ones, were confirmed by analysis of the samples with a Thermo Scientific iCAP RQ ICP_MS operating in kinetic energy discrimination (KED) mode to quantify Cu at *m*/*z* 63 and 65.

### 2.8 Cell Culture and Treatments

The immortalized human fetal osteoblast cell line hFOB1.19 was grown in a complete DMEM/Ham’s F12 medium supplemented with 10% fetal bovine serum (FBS), 2 mM sodium pyruvate (Gibco), 50 IU/mL penicillin, and 50 μg/mL streptomycin. For the cell treatment, the cells were plated and treated for 4 days with Hap and derivatives in starvation medium (DMEM/Ham’s F12 medium supplemented with 1% FBS, 2 mM sodium pyruvate, 50 IU/mL penicillin/streptomycin). To investigate the role of Cu^2+^ in osteoblast differentiation, the cells were simultaneously treated with 2,9-dimethyl-4,7-diphenyl-1,10-phenanthroline disulfonic acid (BCS) (50 μM), an extracellular chelator of Cu^2+^.

### 2.9 Cellular Cytocompatibility, Proliferation, and Morphology Analysis of Raw 264.7

The effect of Hap and Hap-Car treatments on cell viability was analyzed with the MTT method, as previously described.^97^ The cell proliferation and morphology were analyzed and quantified through a label-free approach using the IncuCyte SX1 (Serial Number: IC 60068) live cell imaging system.

### 2.10 Alizarin Red Staining

The extent of extracellular matrix mineralization was evaluated on day 4 of differentiation by Alizarin Red S staining.

### 2.11 Alkaline Phosphatase Activity (ALP) Assay

The osteogenic activity was evaluated by an ALP assay kit (Colorimetric; Abcam, ab83369) according to the manufacturer’s instructions.

### 2.12 Enzyme-Linked Immunosorbent Assay (ELISA)

Medium samples were collected after 4 days of treatments, and the amounts of BDNF and BMP-2 secreted in the cell culture medium were quantified using the Human BDNF Simple Step ELISA kit (Abcam (MA, USA); ab212166) and Human BMP2 ELISA kit (Thermo Fisher Scientific, EHBMP2), according to the manufacturer’s instructions.

The level of osteocalcin release was determined from the cell culture medium samples using a direct ELISA assay. The level of VEGF release was analyzed in the cell culture medium as previously described.^98^

### 2.13 Immunocytochemistry

To study BMP-2 expression, immunocytochemistry analysis was carried out according to the method described in a previous paper.^99^

### 2.14 Protein Lysate Preparation and Immunoblotting

Expression analysis of both the osteogenic marker BMP-2 and the main copper homeostasis players (Ctr1, CCS, ATP7B) was carried out by Western blotting as described in previous papers.^100^

### 2.15 Long-Term Maintenance of Hap and Hap-Car in Culture Medium

Hap, Hap-Car10:1, Hap-Car2:1, and Hap-Car1:1 (0.05 mg/mL) were incubated in cell culture starvation medium for 2 weeks in a humidified incubator at 37 °C in the absence of the hFob1.19 cells. After that, Hap, Hap-Car 10:1, Hap-Car 2:1, and Hap-Car 1:1 were centrifuged, transferred into clean Eppendorf tubes with PBS, centrifuged again, and then added to hFob1.19 cells in a fresh starvation medium for 4 days. The effects of these Hap and all HapCar derivatives were investigated, evaluating again their cytocompatibility by the MTT assay and BMP-2 expression level by immunoblotting.

### 2.16 Data and Statistical Analysis

Results were expressed as mean ± standard deviation of at least three experiments performed in triplicate. One-way analysis of variance (ANOVA) followed by Tukey’s posthoc test was used for comparisons between all groups, whereas Student’s *t* test was used to compare the means between two groups with or without BCS. All statistical analyses were performed with GraphPad Prism 6.0 software (GraphPad Software Inc., La Jolla, CA, USA). Differences were considered to be significant at *p* values of <0.05.

## 3 Results

### 3.1 Synthesis and Characterization of the Compounds

#### 3.1.1 Synthesis

Hap-Car derivatives were synthesized by coprecipitation of calcium, Car, and phosphate solutions (Scheme 1 in the SI). As reported in the literature, various molecules of biological origin (amino acids, peptides) as modulators of biomineralization may affect the crystallization of Hap.^101,102^

The three different derivatives were obtained by varying the Ca^2+^:Car ratio (1:1, 2:1, and 10:1). After mixing, the pH was maintained constant at 7.4 to improve the crystallization and aging of the Hap-Car derivatives^103^ (Supporting Information, Scheme 1).

#### 3.1.2 X-ray Diffraction Analysis

The atomic crystalline nature of the samples (Hap, Hap-Car1:1, Hap-Car2:1, and Hap-Car10:1) was investigated using X-ray diffraction (XRD) (Figure 1).

**Figure 1:**
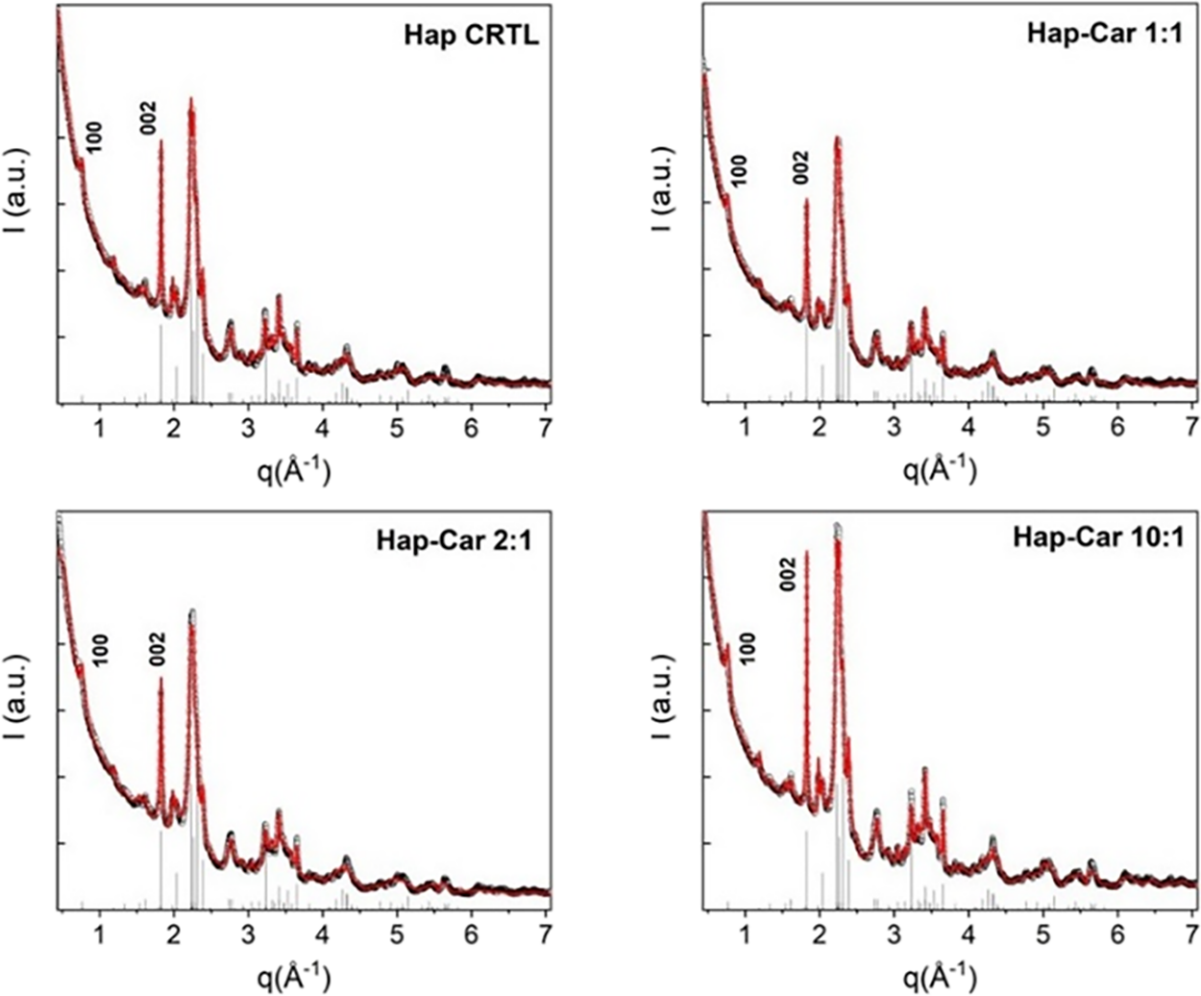
XRD profiles (experimental: dotted black, fitted: red full lines) of the Hap, Hap-Car1:1, Hap-Car2:1, and Hap-Car10:1 versus scattering vector *q* = 4π sin­(*q*)/λ. Vertical bars mark the Bragg peak positions of the identified hexagonal hydroxyapatite phase (Ca_5_(PO_4_)_3_(OH), ICSD #187840). Two relevant orthogonal reflections, the (100) and (002), are marked.

The XRD profiles were examined by using a Rietveld-based quantitative method. The crystal structure responsible for the peak positions and relative intensities was initially identified as hexagonal hydroxyapatite (Ca_5_(PO_4_)_3_(OH), ICSD #187840) with the aid of QualX2.0 software.^104^ Subsequently, the FullProf Rietveld program [recent developments of the program FULLPROF, Juan Rodriguez-Carvajal, NEWSLETTER No. 26, December 2001, http://www.iucr.org/iucr-top/comm/cpd/Newsletters/] was used to fit the whole profiles, using spherical harmonics (ISizeModel = 19) to describe the inhomogeneous peak broadening of the XRD reflections, based on a phenomenological model derived from a modified Scherrer formula. The cell size and peak width were treated as free parameters, while the background was interpolated rather than refined. The instrumental resolution function was determined by analyzing the diffraction pattern of the LaB6 NIST standard recorded under the identical experimental conditions.

The Rietveld analysis of the XRD data allowed us to determine the unit cell size and crystalline domains, including average apparent size and standard deviation, summarized in Table 1 along with the goodness of fit statistical indicator (GoF), which takes the value of 1 for an ideal fit. The apparent sizes along the crystallographic directions (002) and (100) are also reported.

**Table 1:** Results of Rietveld Analysis of the XRD Data

	unit cell						
samples	*a* = *b* (Å)	*c* (Å)	average apparent size (Å)	standard deviation (anisotropy) (Å)	apparent size along 002 (Å)	apparent size along 100 (Å)	goodness of fit
Hap-Car 1:1	9.447 ± 0.005	6.875 ± 0.005	103 ± 1	27 ± 1	185 ± 1	81 ± 1	0.95
Hap-Car 2:1	9.449 ± 0.005	6.875 ± 0.005	89 ± 1	25 ± 1	164 ± 1	68.5 ± 1	1.3
Hap-Car 10:1	9.439 ± 0.005	6.875 ± 0.005	116 ± 1	46 ± 1	276 ± 1	83 ± 1	1.2
Hap	9.443 ± 0.005	6.876 ± 0.005	118 ± 1	37.5 ± 1	239.5 ± 1	89 ± 1	0.92

Overall, our results indicate that the three different Hap-Car derivatives obtained by varying the Ca^2+^:Car ratio (1:1, 2:1, 10:1) present different structural features; in particular, Hap-Car10:1 shows an average apparent size and an anisotropy value more similar to Hap with respect to the values found for Hap-Car2:1 and Hap-Car1:1, suggesting an effect of Car in the Hap mineralization. The Hap composite with the minimal crystalline domains is Hap-Car2:1, along both crystallographic directions. In terms of trends, Hap-Car10:1 > Hap-Car1:1 > Hap-Car2:1, i.e., from the highest to the lowest domain size.

#### 3.1.3 FT-IR Analysis

The functional groups of the samples were identified by using FT-IR spectroscopy. The FT-IR spectra of all three Hap-Car derivatives are almost similar due to the presence of phosphate and hydroxyl groups typical of Hap,^105^ which produce peaks at fixed wavelengths (Figure 2).

**Figure 2:**
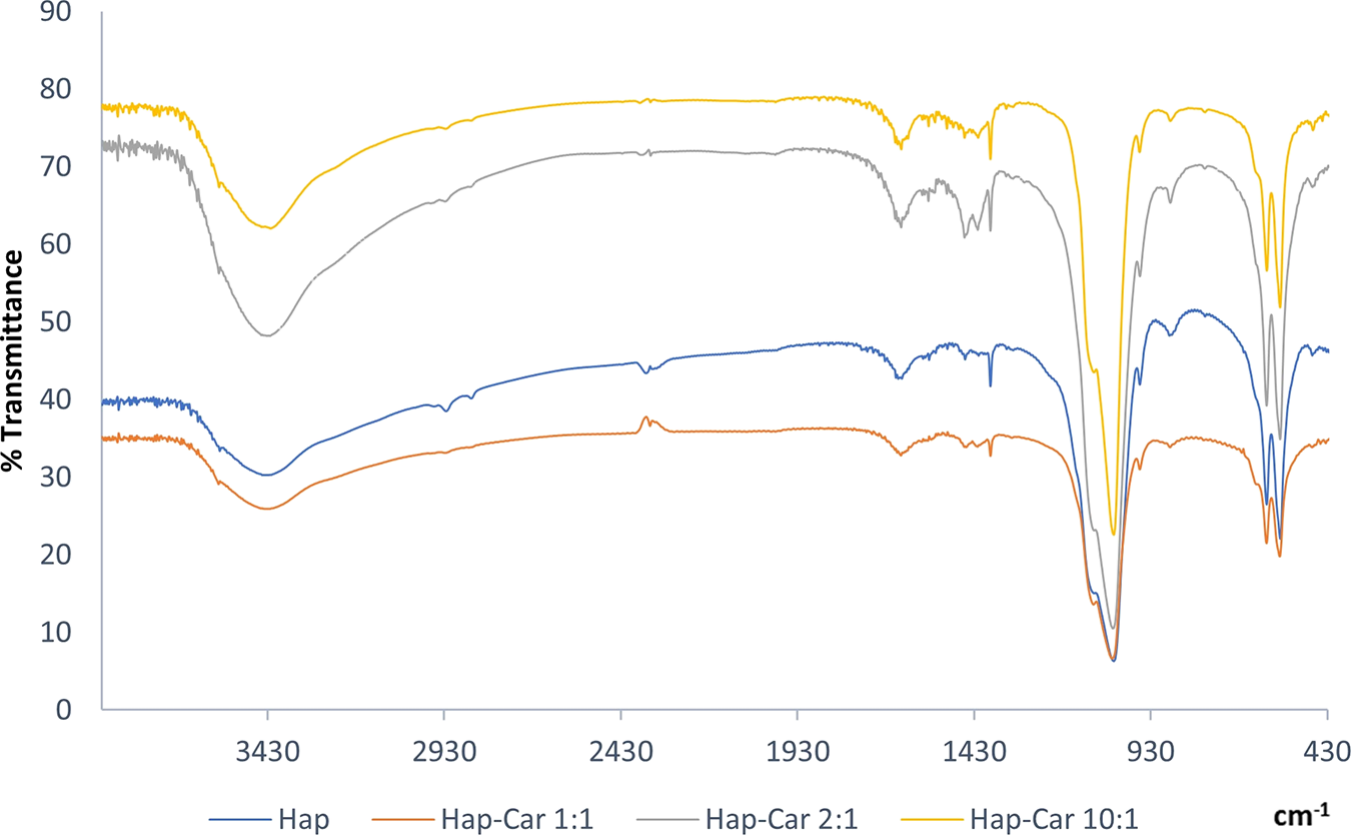
Comparison between FT-IR spectra of Hap and Hap-Car derivatives (Hap-Car1:1, Hap-Car2:1, Hap-Car10:1).

In particular, the FT-IR spectra of all synthesized Hap powders show the characteristic peaks at 1636, 1079, 1036, and 960 cm^–1^, which are due to the phosphate and carbonate stretching vibrations, while the peaks at 602 and 562 cm^–1^ are related to the bending vibrations of the phosphate groups. In addition, the broad band at 3418 cm^–1^ can be assigned to the stretching vibrations of the hydroxyl groups, as previously reported in the literature.^106^ The peaks at 1449 and 1414 cm^–1^, which can be assigned to the vibrations of the carbonyl groups, appear more evident in the spectrum of Hap-Car2:1.

#### 3.1.4 SEM Analysis

Sample morphology was investigated by means of SEM at 2 kV, acquiring secondary electrons with a probe current of 100 pA (Figure 3, Figure S1). At this magnification (≈5000×), samples Hap-Car1:1 and Hap-Car2:1 exhibit a high amount of submicrometer particles, while in samples Hap-Car10:1 and Hap, we observe 2D structures with sizes in the range of 1–20 μm. Figure 3 shows the same samples at higher resolution and magnification. In this condition, the nanostructured surface along the four specimens is also visible. In particular, the 2D surface in Hap-Car10:1 and Hap is not flat, but it is characterized by nanoparticles and nanovoids of the order of few tens of nanometers.

**Figure 3:**
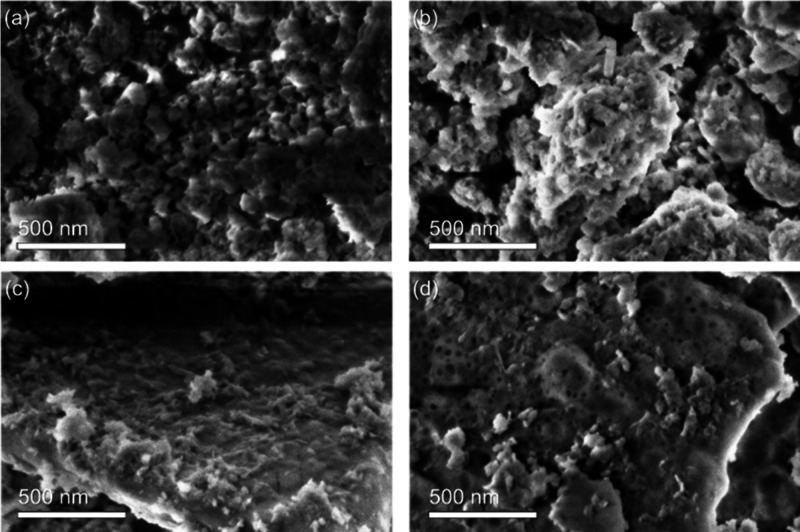
SEM comparison between Hap-Car derivatives at high resolution: (a) Hap-Car 1:1, (b) Hap-Car 2:1, (c) Hap-Car 10:1, and (d) Hap.

#### 3.1.5 Carnosine Loading

The loading amount of Car entrapped in the Hap-Car derivatives was determined by a spectrofluorimetric method. The method was based on the derivatization of primary amine with OPA reagent and adapted to perform the measurements in a 384-well microplate.^106^

The calibration curve was constructed with different standard solutions of Car (*n* = 10, linear range 0.2–40 μM, R2 0.99). The calculated loading values are listed in Table 2.

**Table 2:** Car Loading Values for Hap Derivatives Calculated by the OPA Fluorimetric Method

	% Car loading
Hap-Car 1:1	0.86(±2)
Hap-Car 2:1	1.78(±3)
Hap-Car 10:1	0.12(±3)

The Hap-Car derivatives show peptide loading values between 0.1 and 1.8%. Car loading percentages are in line with the apparent size values obtained by XRD analysis and follow the same trend (Hap-Car10:1 < Hap-Car1:1 < Hap-Car2:1, i.e., the highest Car loading gives the lowest domain size). With the aim of determining the amount of Car trapped in Hap, the three Hap-derivatives were incubated in phosphate buffer (10 mM, pH 7.4) and the peptide percentage was calculated at different times. The Car level does not change in Hap-Car10:1, suggesting a stable incorporation in Hap, while Hap-Car1:1 and Hap-Car2:1 show a continued decrease in the peptide level, indicating a less stable incorporation in Hap that favors a significant release in a solution of Car (Figure 4). This different behavior appears consistent with the different morphologies and structures put in evidence by the XRD and SEM results.

**Figure 4:**
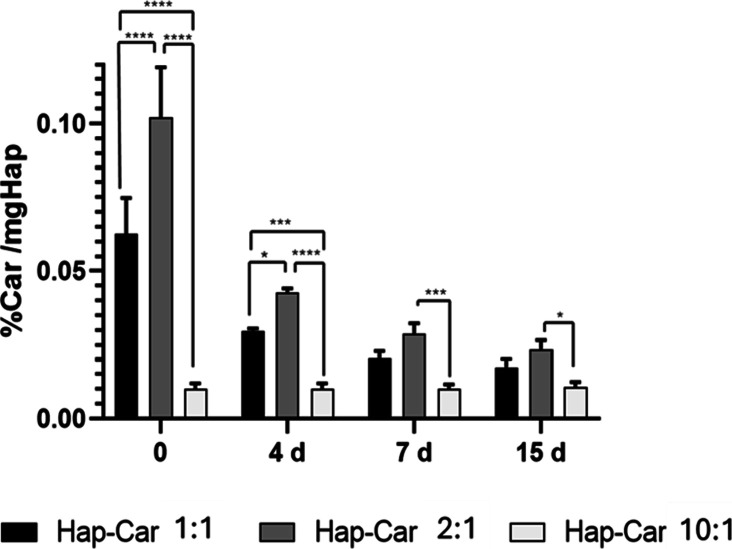
Percentage of Car trapped in Hap derivatives as a function of incubation time. Hap-Car1:1, Hap-Car2:1, and Hap-Car10:1 were incubated at different times, *t* = 0, *t* = 4, *t* = 7, and *t* = 15 days, and the percentages of remaining carnosine in Hap were determined. Hap-Car1:1, black bar; Hap-Car2:1, gray bar; Hap-Car10:1, light gray bar. (**** *p* < 0.0001, *** *p* < 0.001, *p < 0.05; *n* = 3).

#### 3.1.6 Quantification of the Copper­(II) Content in Culture Media Supplemented with Serum and in Hap and Hap-Car Derivatives

Different cell types require essential biometal ions in bioavailable form to grow, proliferate, and differentiate in culture. These elements include those in the d-block, mainly iron, copper, and zinc.

The serum usually added as a supplement significantly contributes to the metal ion composition of the complete cellular medium,^106^ while further amounts of trace elements can come from the presence of these biometals in buffers.^107^ Despite the increased interest in metallostasis,^108^ the determination of the concentrations of these metal ions in culture medium and serum is often neglected.

Consequently, ICP-OES measurements were carried out to determine the copper contents of both the cell culture media and the serum used to supplement the media. The copper amount in the culture medium utilized for hFOB1.19 cell growth, DMEM/F12, was 0.02 ± 0.01 μM and rose to 0.25 ± 0.03 μM following the addition of 10% FBS. For the hFOB1.19 treatment, the cells were in the same medium but with the addition of 1% FBS (copper content 0.043 μM) and were treated for 4 days with different concentrations of Hap, Hap-Car10:1, Hap-Car2:1, and Hap-Car1:1. This incubation in complete medium also modified the copper content in Hap and in its Car entrapped derivatives whose concentration changed after 4 days from 2.08 ± 0.09 to 1.24 ± 0.03 ng/mg for Hap, from 0.93 ± 0.03 to 1.18 ± 0.01 ng/mg for Hap-Car10:1, from 1.67 ± 0.01 to 1.51 ± 0.02 ng/mg for Hap-Car2:1, and from 1.00 ± 0.01 to 2.76 ± 0.02 ng/mg for Hap-Car1:1. This behavior is related to the minor metal affinity of the phosphate oxygen donor of Hap in comparison with that of Hap-Car scaffolds due to the chelating ability of the nitrogen donor atoms of the entrapped peptide. The differences among the Hap-derivatives can be attributed to the different amounts of Car present in the three scaffolds and their diverse release rates of dipeptide, as highlighted above.

### 3.2 Biological Studies

#### 3.2.1 Hap and Hap-Car Cytocompatibility and Cell Proliferation Evaluation

For the determination of the hFOB1.19 cell viability, different concentrations of Hap and Hap-Car (0.05 or 0.1 mg/mL in hydroxyapatite) were added to the culture medium (Figure 5). All Haps do not show cytotoxicity at 24 h, while a slight reduction in cell viability (10–20%) is found at 4 d (Figure 5A, B).

**Figure 5:**
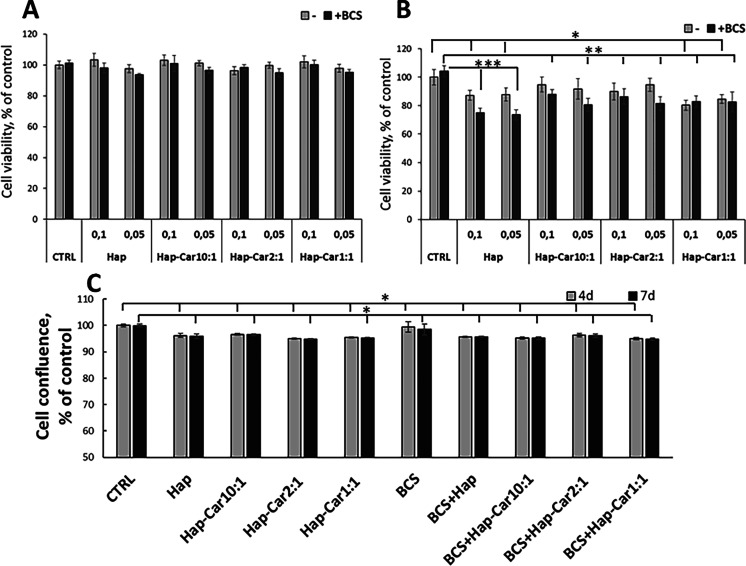
hFOB1.19 cell viability and proliferation. Cells were treated with Hap, Hap-Car10:1, Hap-Car2:1, and Hap-Car1:1 in the presence or absence of BCS (50 μM). Cell viability was measured at 24 h (A) and 4 days (B) by the MTT assay. (C) Cell proliferation was analyzed with the IncuCyte instrument during 7 d. The percentage of cell confluence was calculated at 4 d and 7 d as the total occupied area of hFOB1.19 cells vs time 0 by Incucyte Base Analysis with “Artificial Intelligence” mask for cell detection (for whole cell growth curves, see Figure S2; for other parameters, refer to Table S1). The data points and error bars represent mean ± SD. Statistical significance is indicated as *p < 0.05, **p < 0.01, ***p < 0.001.

Being aware of the presence of copper­(II) ions in the complete medium, the influence of copper on the viability and proliferation was also studied. The culture medium was first incubated with the copper chelator 2,9-dimethyl-4,7-diphenyl-1,10-phenanthroline disulfonic acid (BCS) overnight, and then the cells were exposed to Cu^2+^ deprived-medium with hydroxyapatite or its derivatives added to it. BCS does not affect cell viability after 24 h (A) and 4 days (B) of incubation (Figure 5), apart from Hap, which exhibits less cytocompatibility compared to Hap-Car derivatives (Figure 5B). Based on these results, subsequent experiments were performed using Hap 0.05 mg/mL alone or Hap-Car. To discriminate between the antiproliferative and cytotoxic effects, we performed a real-time cell proliferation analysis of the hFOB1.19 cells at 80% confluence using the IncuCyte Live Cell Analysis System tool. Images of the cells acquired every 6 h were analyzed using artificial intelligence (AI). Cells after Hap and Hap-Car derivative treatments (Figure 5C, Figure S2) continue to proliferate for the first 24 h and then reach a plateau, indicating stable viability and the absence of late-onset cytotoxic effects.

The deprivation of copper by BCS does not affect cell proliferation curves after 4 days of incubation but slightly decreases cell confluence after 7 days; however, this reduction was not statistically significant (Figure S2). As shown in the cell growth curves, Hap-Car1:1 in the presence and absence of BCS shows reduced cell proliferation with slight toxicity after 48 h (Figure 5C, Figure S2A, B). The arrest of cell proliferation observed upon Hap-Car derivative treatments is consistent with the reduction in cell viability assessed by MTT, supporting the concept that cells typically exit the cell cycle and cease proliferating upon undergoing differentiation. This result may be related to a metabolic switch associated with Hap-induced cell differentiation, as well as to the well-known effect of copper on the proliferation and differentiation processes.^109^

#### 3.2.2 Hap-Car Promotes Osteogenic Differentiation of hFOB1.19, Affecting Cell Morphology, Mineralization, and Alkaline Phosphatase Activity

In order to analyze the effects of Hap and Hap-Car derivatives on hFOB1.19 differentiation, the cell morphology and some markers of osteogenesis such as mineral deposition and ALP activity were assessed. Cells maintained healthy morphology, reached confluence, and showed no signs of structural damage, indicating that the treatments do not exert harmful effects over time (Figure S3). Alizarin Red-stained calcium deposits at day 4 are consistent with the results of proliferation analysis (Figure 5). Cells upon treatment with Hap and Hap-Car derivatives (Figure 6 A, B) are uniformly elongated with calcium (reddish spot) deposition. The addition of BCS induces a lower deposition of calcium than cells treated with single substances. Interestingly, the quantitative analysis of Alizarin Red staining of calcium deposits shows a Ca^2+^:Car ratio increase; Hap-Car10:1 shows the highest Alizarin staining (191% ± 6) in comparison with both the other two Hap derivatives (Hap-Car2:1, 151% ± 4; Hap-Car1:1, 132% ± 1) and Hap (147% ± 4) (Figure 6C).

**Figure 6:**
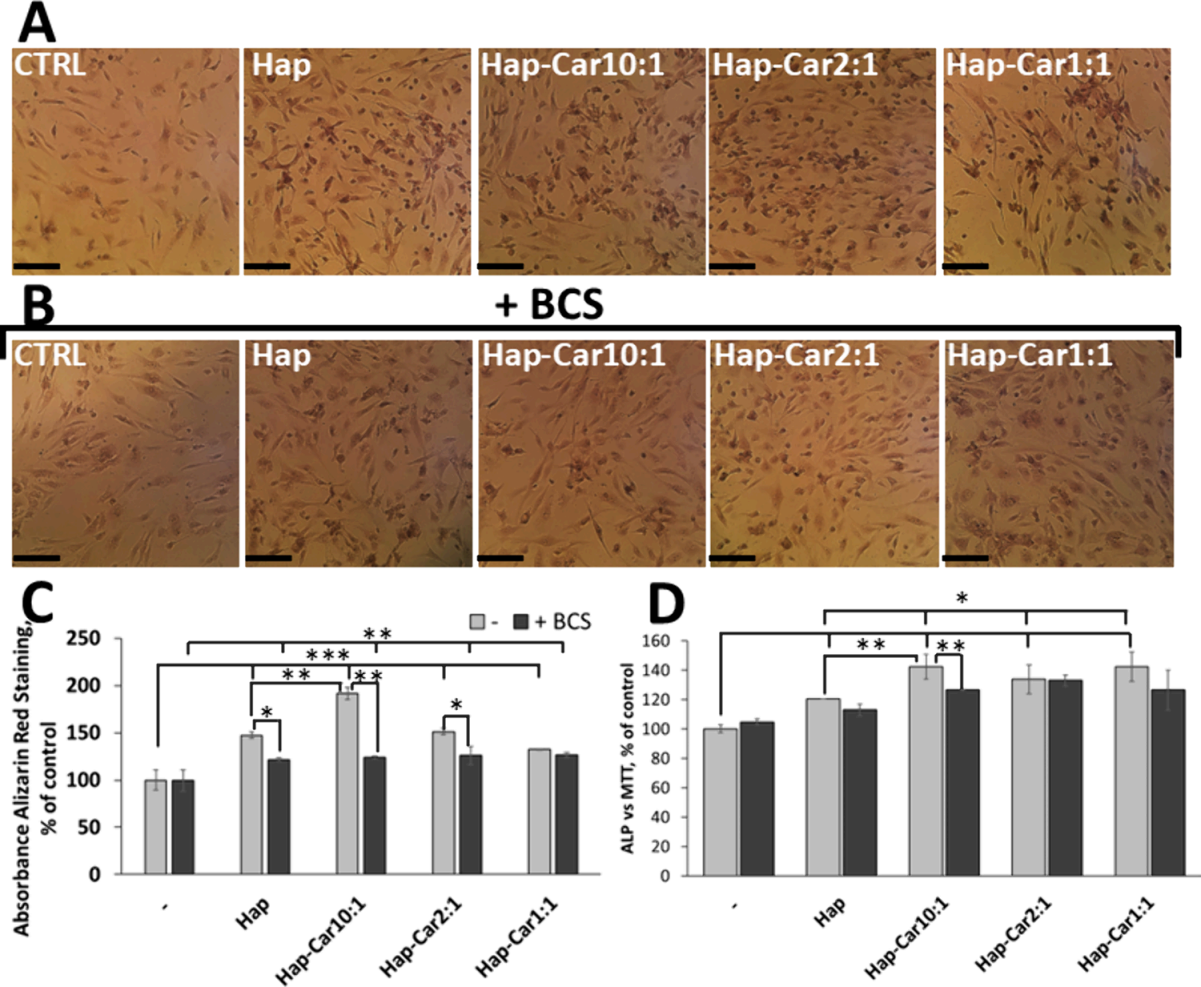
Hap-Car promotes biomineralization and enhances ALP activity. (A–C) Alizarin Red staining and (D) ALP activity in hFOB1.19 cells treated with 0,05 mg/mL Hap, Hap-Car10:1, Hap-Car2:1, and Hap-Car1:1 with or without BCS (50 μM) at day 4. (A, B) Representative photos of stained hFOB1.19 cells in the presence (B) and absence (A) of BCS visualized using a phase contrast inverted microscope at 20× magnification. Mineralization indicated by calcium deposition appears as reddish spots. (Scale bar: 131 μm). (C) The results of quantitative analysis of Alizarin Red staining and (D) ALP activity are presented as mean ± SD. Statistical significance is indicated as *p < 0.05, ** *p* < 0.01, and *** *p* < 0.01.

ALP activity was tested as an early biomarker of osteoblast differentiation. Hap and its Car-derivatives induce significant differences in ALP activity, already at day 4 (Figure 6 D). Hap enhances ALP activity up to 120% ± 1 compared to untreated cells. Based on the Ca^2+^:Car ratio, the 10:1, 2:1, and 1:1 Hap derivatives significantly increase ALP activity to 142% ± 9, 134% ± 10, and 142% ± 10, respectively. When BCS was added, a significant reduction of ALP activity was observed mainly for Hap-Car10:1 (127% ± 1), highlighting the role of copper, in keeping with the results found for biomineralization.

#### 3.2.3 Hap and Its Car Derivatives Affect Expression of Osteoblastogenesis Markers and Induce BDNF and VEGF Release in a Copper-Dependent Manner

Next, we evaluated the production of BMP-2 to additionally confirm that Hap and its Car derivatives exhibit bone regenerative properties by promoting the differentiation of osteoblasts. The expression level of BMP-2 increases after incubation with Hap, and the same behavior is shown by its Car derivatives (Figure 7A, B). Hap-Car10:1 triggers a higher BMP-2 expression (195% ± 6) with respect to the other derivatives (Hap-Car2:1, 145% ± 4; Hap-Car1:1, 150% ± 9) and Hap (136% ± 5). BCS significantly reduces the BMP-2 expression caused by Hap-Car10:1 (117% ± 16), Hap-Car2:1 (120% ± 16), Hap-Car1:1 (118% ± 2), and Hap (129% ± 7), highlighting the role of copper.

**Figure 7:**
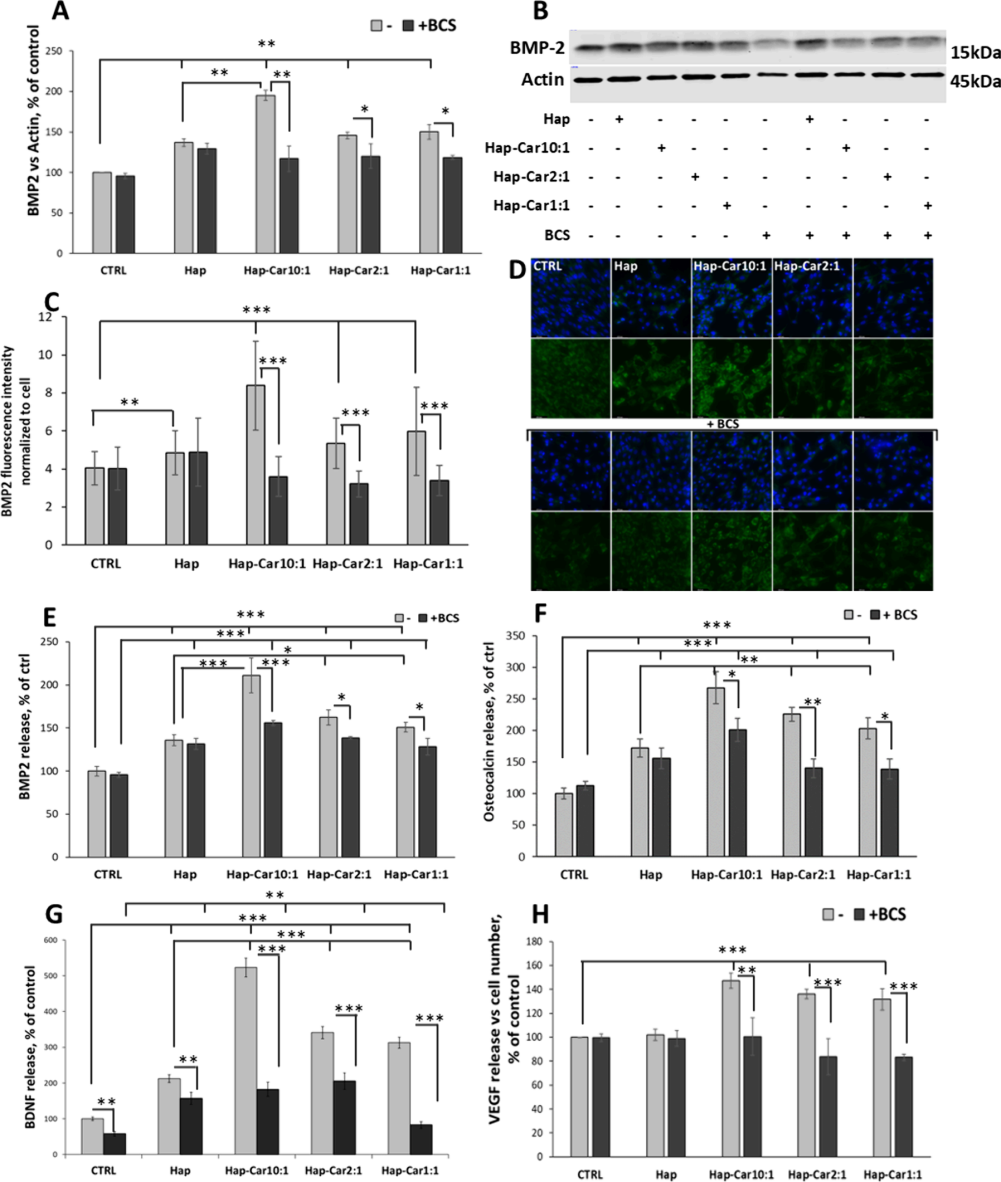
Effect of Hap-Car on osteogenic markers and trophic factors. hFOB1.19 cells were stimulated with Hap (0.05 mg/mL), Hap-Car10:1, Hap-Car2:1, and Hap-Car1:1 with or without 50 μM BCS for 4dd. Densitometric analysis (A) and representative Western blot image (B) of BMP-2 expression levels. The expression level of BMP-2 is reported as % of control of ratio over Actin. (C) The quantitative analysis of fluorescence for BMP-2 expression was analyzed using ImageJ Software1.53 and is expressed as fluorescence intensity normalized with the number of cells per photo. (D) Representative images of hFOB1.19 cells acquired by fluorescence microscopy after 4dd treatment. The cells were incubated with anti-BMP-2 antibody (green) and nuclear dye Hoechst33348 (blue). Scale bar 65.8 μm, magnification 40×. The extracellular release of BMP-2 (E), osteocalcin (F), BDNF (G), and VEGF (H) is reported as a ratio over the cell number calculated by Incucyte software and reported as % of control. Data are expressed as mean ± SD. Statistical significance is indicated as *p < 0.05; ** *p* < 0.01, and *** *p* < 0.001.

These results were also confirmed by immunofluorescence (Figure 7C, D). All treatments induce a marked expression of BMP-2. The intensity of fluorescence indicating the BMP-2 expression shows significant differences between Hap and its Car derivatives. BCS addition induces a marked decrease in BMP-2 expression (Figure 7C, D). These results were also confirmed by both immunofluorescence (Figure 7C, D) and BMP-2 release (Figure 7E), with a marked increase in endo- and extracellular BMP-2, especially with Hap-Car. Addition of BCS reduces these levels. The same trend for BMP-2 is observed with osteocalcin release. It increases with all treatments, whereas in the presence of BCS, its release is inhibited.

Furthermore, Hap and its Car-derivative treatment significantly increased the release of BMP-2 (Figure 7E) and osteocalcin (Figure 7F) compared to an untreated control. Secretion of BMP-2 increases in the presence of Hap up to 135% ± 6, while its Car-derivatives show larger effects (Hap-Car10:1 – 211% ± 20, Hap-Car2:1 – 162% ± 9, Hap-Car1:1 – 150% ± 6). The extracellular copper chelator BCS reduces BMP-2 secretion in all the Hap-Car derivative treatments.

Osteocalcin is used as a late marker of osteogenesis. Its production is stimulated by BMP-2 ^110,111^ and is consistent with the features of mature bone.^112^ Incubation of cells in the presence of Hap results in increased secretion of this protein. The highest effect is observed in the presence of Hap-Car10:1 (268% ± 26), while it decreases with rising Ca^2+^:Car ratio (225% ± 11 for Hap-Car2:1 and 203% ± 17 for Hap-Car1:1). The cells treated with BCS and Hap-Car derivatives exhibit a significant reduction in the level of osteocalcin release.

Our results indicate that Car entrapped in Hap increases the capacity of Hap in activating the osteogenic markers BMP-2, osteocalcin, ALP activity, and calcium deposits in copper-dependent mode, stressing again the copper role in these processes.

The osteogenic niche affects many processes involved in osteogenesis, differentiation, and bone regeneration through the secretion of cytokines, chemokines, and growth factors as neurotrophins.^113^ BDNF synthesis occurs in several peripheral tissues, including bone, skeletal muscle, white adipose tissue, and liver for maintaining systemic metabolism by autocrine/paracrine/endocrine functions,^114,115^ and it also promotes hBMSC osteogenesis *in vitro* and *in vivo*.^93^ Recent results indicate that Car induces expression and secretion of BDNF in PC12 ^116^ and U-87 MG^117^ cells, suggesting that the dipeptide activates these cells through increased production of neurotrophins.

We found that by day 4 post-treatment, BDNF is widely released by Hap-Car-treated hFOB1.19 compared with Hap alone, and the amount of released BDNF is inversely dependent on the Ca^2+^:Car ratio (Figure 7G). The most effective Hap-Car derivative to elicit a response in cells is Hap-Car10:1 (523% ± 62), followed by Hap-Car2:1 (341% ± 36) and Hap-Car1:1 (313% ± 24). BCS addition strongly inhibits the effect of all compounds on BDNF release (157% ± 38 for Hap, 182% ± 28 for Hap-Car10:1, 205% ± 13 for Hap-Car2:1, and 83% ± 18 for Hap-Car1:1) (Figure 7G).

The effect of Hap-Car derivatives on trophic factors is also evident on VEGF release; Hap-Car derivatives promote VEGF release, while Hap does not affect the extracellular levels of VEGF. Similar to BDNF results, this effect is inversely dependent on the Ca^2+^:Car ratio, with Hap-Car10:1 (147% ± 6) more effective than other derivatives (136% ± 6 for Hap-Car2:1; 132% ± 22 for Hap-Car1:1) (Figure 7H). The involvement of copper in the effects mediated by Hap-Car derivatives is also evident on VEGF release, which is drastically blocked by BCS (Hap-Car10:1 – 100% ± 8; Hap-Car2:1 – 84% ± 1; Hap-Car1:1 – 83% ± 5) (Figure 7H). Hap-Car derivatives trigger a neurotrophic-based osteogenic mechanism supported by copper, which exerts a key role in the trophic and angiogenic ability of BDNF and VEGF.

#### 3.2.4 Long-Term Maintenance of Hap and Hap-Car in Culture Medium Slightly Affects Their Osteogenic Properties

The bioviability and osteogenic capability of Hap and its derivatives with Car maintained in the cell culture medium alone for 2 weeks were investigated through the effect on cytotoxicity, as well as by measuring the expression level of the osteogenesis marker BMP-2 (Figure 8).

**Figure 8:**
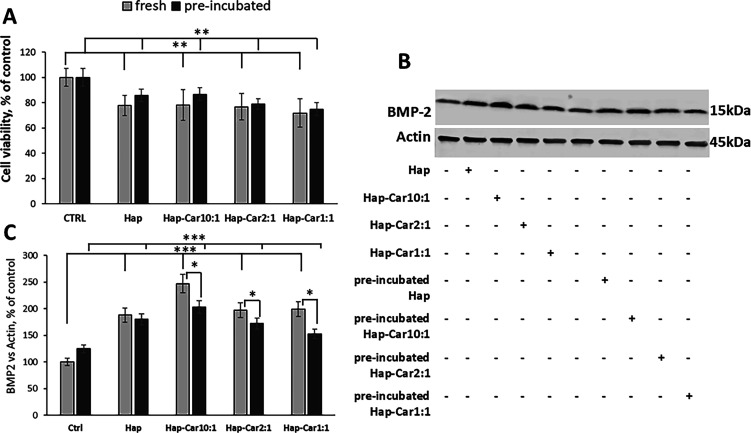
Effect of long-term maintenance of Hap and Hap-Car composites in culture medium on bioviability and osteogenic activity. Hap and Hap-Car derivatives were maintained in a complete medium without cells for 2 weeks. Cells were treated with 0.05 mg/mL of Hap, Hap-Car10:1, Hap-Car2:1, and Hap-Car1:1 (preincubated with medium for 2 weeks or fresh) for 4 d. (A) Cell viability was measured by the MTT assay. (B) Densitometric analysis and representative Western blot (C) of the BMP-2 expression level. Data are expressed as mean ± SD. Statistical significance is indicated as *p < 0.05, ** *p* < 0.01, and *** *p* < 0.001.

All the substances kept in medium for 2 weeks and those freshly prepared do not show significant differences in their bioviability (Figure 8 A). BMP-2 levels increase after treatment with both Hap and its composites, but the effect is less marked for the preincubated samples. (Figure 8B, C). Unlike Hap, Hap-Car derivatives show a significant statistical difference between preincubated and fresh samples. The release of entrapped carnosine in Hap during preincubation of Hap-Car composites gives reason for this behavior. Our results indicate that Hap and Hap-Car derivatives retain their osteogenic properties also after incubation in the cell medium for 2 weeks.

#### 3.2.5 Hap and Hap-Car Derivatives Affect Copper Homeostasis

Our results indicate that Hap and Hap-Car derivatives induce osteogenic and angiogenic processes in copper-driven mode; this behavior of hydroxyapatite and its synthesized composites prompted us to study their influence on copper homeostasis in 48 h treated hFOB1.19 cells (Figure 9).

**Figure 9:**
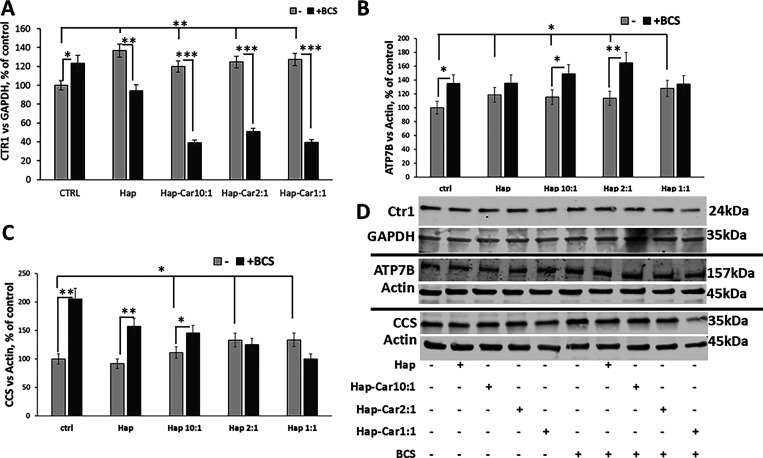
Hap and its Car derivatives affect copper homeostasis during osteoblast differentiation. Densitometric analysis of Ctr1 (A), ATP7B (B), and CCS (C) expression levels and representative Western blot images (D) and representative Western blot images (F) of Ctr1 (A), ATP7B (B), and CCS (C) expression levels in hFOB1.19 cells treated for 48 h. The expression levels of Ctr1, ATP7B, and CCS are reported as the ratio over GAPDH or actin. Data are expressed as mean ± SD. Statistical significance is indicated as *p < 0.05, ** *p* < 0.01, and *** *p* < 0.001.

Ctr1 protein expression was used as a sensor to evaluate the trafficking of copper. After the treatments, the expression of Ctr1 increases (30% vs untreated cells) (Figure 9A) as expected, when the extracellular environment (the complete culture medium) is deprived of copper. Differently, the presence of BCS, whose chelating ability for the metal ion could compete with that of hydroxyapatite with and without trapped carnosine, induces a drastic decrease of Ctr1 expression (Figure 9A), highlighting that the investigated compounds affect copper homeostasis.

Hap and its derivatives impact ATP7B and CCS expression at a cytoplasmic level, inducing a slight increase, while BCS causes a further enhancement of ATP7B and CCS expression (Figure 9B, C). These findings suggest that Hap-Car derivatives tune intracellular copper ion homeostasis, affecting metal ion trafficking.

## 4 Discussion

Taken together, our findings indicate that Hap-Car composites promote the expression and release of some trophic, angiogenic, and osteogenic factors, strengthening the regenerative capacities of Hap. Car contribution to these increased effects is copper dependent due to the dipeptide chelating ability of Cu^2+^ present in the complete culture medium at submicromolar concentrations. Moreover, the XRD data suggest an effect of Car in the Hap mineralization process. In fact, it is known that amino acids, short peptide sequences, and small molecules have a strong effect on crystallization processes.^118–121^ The major beneficial effect of Hap-Car10:1 in comparison with that displayed by the other two Hap-Car derivatives highlights the role played by both the more microstructural and morphological similarity to Hap shown by Hap-Car10:1 and the more stable entrapment of the dipeptide in the hydroxyapatite structure.

Recently, the role of nanohydroxyapatite and its composites in heavy metal ion (HMI) decontamination has been reported, highlighting that most of the HMIs are adsorbed by Hap through surface complexation and ion exchange.^122^ To solve some issues in the n-Hap absorption effects on the HMI,^123,124^ new Hap composites have been synthesized combining n-Hap with other materials in order to achieve functional complementation or to impart new properties to n-Hap, including chelating molecules for metal ion removal.^125,126^ Most of the Hap metal absorption studies have been performed in a simple laboratory environment, while adsorption effects in real complex environments have been less studied; our findings show that Hap can interact with copper in a biological environment such as that represented by the complete culture medium. The Hap copper binding is evidenced by the decreased beneficial capacities shown in the presence of the competitive ligand BCS.

Previously, different reports have highlighted Hap and its different composite abilities to stimulate the expression and activity of some crucial factors (ALP, type I collagen, OSC, etc.) for efficient bone regeneration. It is noteworthy that although the cells experienced a culture medium supplemented with serum similar to the complete medium employed in our assays, the role of copper was overlooked.^127–130^

In recent years, different studies have highlighted the involvement of trace metal ions in bone health and regeneration,^130,131^ showing their abilities to support bone growth, modeling, and remodeling.^46,132,133^ Approximately 70% of the copper in our bodies is localized in our muscles, cartilages, and bone tissues,^134,135^ justifying the relevant role played by copper in regulating^136^ and in stimulating normal bone development as well as in preserving bone and cartilage homeostasis.^137–139^ In this context, the copper­(II) ion emerges to be involved in the diverse steps of some coordinated processes that promote bone tissue regeneration.^140^ In the immune cells, the equilibrium between M1 and M2 macrophages involved in pro-inflammatory and anti-inflammatory responses, respectively,^141^ preserves metabolic homeostasis in the cartilage and synovial environment. The inflammatory activities alter this balance, increasing the M1/M2 macrophage ratio;^142^ M1 polarization heightens inflammation, oxidative stress, and cartilage matrix degradation^143^ Copper stimulates at a concentration below 10 mM^87,144^ the polarization and recruitment of macrophages from the pro-inflammatory M1 phenotype to the anti-inflammatory M2 phenotype,^145^ thereby promoting the repair of bone and cartilage. Copper also contributes to angiogenesis, osteogenesis, and mineralization overlapping processes, which drive the healing of bone tissues.^140^ Furthermore, copper promotes osteogenesis by increasing bone formation by upregulating key bone-related genes including ALP, osteopontin, osteocalcin, collagen type I, and VEGF in endothelial cells. Moreover, copper stimulates the proliferation and activity of osteoblasts,^146^ favoring bone mineralization, which plays a critical role in bone regeneration.^147^ High VEGF levels induced by Cu^2+^ increase the vascularization of bone tissue^148^ essential for bone regeneration by favoring the transport of osteoblasts and bone-forming factors through the vasculature.^149^ VEGF further enhances the expression of BMP-2 in endothelial cells, promoting osteogenic differentiation and mineralization via ALP. BMP-2 can further stimulate VEGF by activating positive feedback responsible of both vascularization and bone formation.^150^ Copper is also a relevant cofactor for different enzymes such as lysyl oxidase (LOX) and Cu,Zn-superoxide dismutase (SOD1), the activities of which can affect osteoclasts and osteoblasts. LOX is a secreted copper-dependent amine oxidase that changes the bone extracellular matrix by catalyzing the covalent cross-linking of collagen and elastin, with consequent enhanced insoluble matrix formation and tensile strength.^151^ The cytoplasmic superoxide anion radical generates bone fragility, decreases mineralization, modifies collagen cross-linking, and causes transcriptional perturbations in the genes related to osteogenesis.^146^ SOD1 catalyzes the disproportionation of the superoxide radical and counteracts the intracellular oxidative stress.^152^

Overall, bone homeostasis requires that copper homeostasis be strictly controlled. It is well known that a complex regulatory mechanism^153^ governs the cellular copper homeostasis both of the tightly bound metal incorporated into proteins and enzymes, the so-called static metallome, where copper plays catalytic and structural roles^152^ and the dynamic metallome that comprises the pool of exchangeable metal ions,^154^ involved in cell signaling.^155^ After its reduction to the Cu^+^ form by the protein STEAP,^156^ copper enters the cell through the membrane protein Ctr1 and binds to three different proteins: antioxidant 1 copper chaperone (ATOX1), Cu chaperone for SOD1 (CCS), and cytochrome c oxidase Cu chaperone (COX17). In the cytoplasm, Cu^+^ is transferred to different organelles, including the Golgi, mitochondria, and nucleus, where it activates topical functions.^157^ Cu^+^ transferred to SOD1 in the cytoplasm and inner mitochondrial membrane via CCS tunes SOD1 activity, decreasing oxidative stress.^158^ COX17 favors the oxidative respiratory chain by transferring Cu^+^ to mitochondrion specific proteins.^159^ Additionally, Atox1 transports Cu^+^ to the Golgi, where it binds to the ATPases, ATP7A/B;^160^ ATP7A mainly promotes metal insertion in copper-dependent enzymes within the Golgi apparatus,^161^ while, when intracellular copper levels rise, ATP7B mediates its excretion through the Golgi-vesicle plasma membrane pathway,^162^ thereby ensuring the maintenance of intracellular copper homeostasis.

Currently, the beneficial contribution of copper is obtained by metal ion doped hydroxyapatite,^90,163^ but the issues of regulating the level of release and localization of the ion incorporated in Hap-based scaffolds can alter copper homeostasis with consequent cellular dyshomeostasis, which impacts osteoblasts, osteoclasts, chondrocytes, and synovial cells, thus affecting the development of bones, cartilage, and synovium.^88,164^

The effect of copper cellular uptake by means of Hap and Hap-Car derivatives on metal homeostasis and hFOB1.19 cell homeostasis was tracked by the changes observed in the expression of copper influx membrane transporter Ctr1 and that of the efflux transporter ATP7B. Ctr1 increases due to metal ion deprivation in the culture medium environment caused by the internalization of the Cu^2+^ entrapped into the scaffold and its Car derivatives, while the ATP7B level enhances to counteract the intracellular increase of copper tuning the metal excretion through the membrane.^165,166^ Thus, the coordinated interplay between the two transporters ensures both copper and cell homeostasis. Furthermore, Hap-Car derivatives increase the intracellular expression of CCS by favoring the SOD1 scavenger activity against the superoxide radical and protecting the osteoblast survival as well as the expression of crucial players of osteogenesis.^152^

## 5 Conclusions

Hap-Car derivatives bind submicromolar copper present in the culture medium showing angiogenic and osteogenic features by the activation of the related protein expression and release; they can be considered good candidates to promote the other beneficial effects of copper involved in bone regeneration, as summarized above.^140^

Furthermore, the Hap-Car composites favor copper cellular uptake, inducing the increase of the high affinity cellular membrane copper transporter 1, Ctr1, which controls the majority of the metal influx^167^ and is required for different trophic factor signals.^168,169^

Recent findings indicate that both CCS and SOD1 are colocalized in the nucleus, suggesting that the transfer of copper to the enzyme by the chaperone can occur also at the nuclear level^170^ and not in the cytosol alone as initially reported.^171^ The presence of CCS in the nucleus has also been related with its involvement in the copper regulation of HIF-1a transcriptional activity^172–174^ that activates VEGF and is involved in LOX expression and activity.^175^ Thus, it is reasonable to suggest that the activation of VEGF release by Hap-Car composites derives from the translocation of CCS at the nucleus, while the inability of Hap to induce both VEGF release and CCS expression can be related to the role played by Car functionalization.

## Supplementary Material



## Notes

Additional experimental details, materials, and methods; Synthesis scheme of Hap and its carnosine derivatives; SEM comparison between Hap-Car derivatives at low magnification (Figure S1); hFOB1.19 cell proliferation curves (Figure S2); Analysis of hFOB1.19 cell morphology (Figure S3) (PDF)
