# Supplementary material for: Carnosine Biofunctionalized Hydroxyapatite Induces Copper-Driven Osteogenesis and Angiogenesis, Strengthening Its Bone Regenerative Capacities
Source: ACS Biomater Sci Eng. 2025 Aug 31;11(10):5878–94. doi: 10.1021/acsbiomaterials.5c00823 (PMC12522101; doi:10.1021/acsbiomaterials.5c00823)
Supplement: Supplementary file 1 [file ab5c00823_si_001.pdf]

**Supplementary Material**

# Carnosine Biofunctionalized Hydroxyapatite Induces Copper-Driven Osteogenesis and Angiogenesis Strengthening its Bone Regenerative Capacities

Irina Naletova[1], Francesco Attanasio[1]*, Teresa Sibillano[2], Barbara Tomasello[1,3], Valeria Lanza[1], Valeria Ciaffaglione[1], Rita Tosto[1], Antonio Mio[4], Warren Cairns[5], Cinzia Giannini[2] and Enrico Rizzarelli[1, 6] *

[1] Institute of Crystallography, National Council of Research, CNR-IC, Via P. Gaifami 18 - 95126 Catania, Italy;
[2] Institute of Crystallography, National Council of Research, CNR-IC, Via G. Amendola 122 - 70126, Bari, Italy;
[3] Department of Drug and Health Sciences, University of Catania, V.le A. Doria 6 - 95125 Catania, Italy;
[4] Institute for Microelectronics and Microsystems, National Council of Research, CNR-IMM, Zona Industriale Strada VIII n. 5, 95121, Catania, Italy;
[5] Institute of Polar Sciences, National Council of Research, CNR-ISP, c/o Campus Scientifico, Università Ca' Foscari Venezia Via Torino, 155 – 30170 Venezia Mestre, Italy;
[6] Department of Chemical Sciences, University of Catania, V.le A. Doria 6 - 95125 Catania, Italy.

* Corresponding authors: Email: francesco.attanasio@cnr.it; erizzarelli@unict.it

**Table of Contents:**

Details of Materials and methods..……………………………………………...……..……..p S2

Synthesis Schema and Supporting Figure

Scheme 1. Synthesis scheme of Hap and its carnosine derivatives ..……… ……….……...p S9

Figure S1. SEM comparison between Hap-Car derivatives at low magnification ..……..…..p S10

Figure S2. hFOB1.19 cell proliferation curves.....……………………………………………...p S11

Figure S3. .Analysis of hFOB1.19 cells morphology ……………………………..…...p S12-13

References..………………………………………………………………………...……..….p S14

MATERIALS AND METHODS

**Reagents**

Calcium chloride dihydrate ($CaCl_2.2H_2O$, ≥99.99% trace metals basis), ammonium phosphate dibasic ($(NH_4)_2HPO_4$, ≥99.99% trace metals basis), L-carnosine (β-alanyl-L-histidine, 99% purity) and ammonium hydroxide ($NH_4OH$) were provided from Sigma Aldrich and used without further purification. Ammonium hydroxide solution 37% was used to adjust the pH during the Hap synthesis. Orthophtaldehyde (OPA) was obtained from Sigma-Aldrich and *N*-acetyl-L-cysteine (NAC) from Acros Organics. Carbonate buffer (0,15 M, pH 10,5) was prepared by dissolving anhydrous sodium carbonate (Sigma-Aldrich) in ultrapure water (Millipore).

All solutions used in experiments were prepared in MilliQ water.

**Synthesis of Hap-Car derivatives**

Hap-Car derivatives were synthesized by co-precipitation of calcium, Car, and phosphate solutions, with varying $Ca^{2+}$: Car ratio (1:1, 2:1, 10:1). An aqueous solution of carnosine (0.5, 0.25 or 0.05 M) and calcium chloride (0.5 M, 10 mL) was added to a solution of ammonium phosphate dibasic (0.3 M, 10 mL) and the resulting suspension was left stirring for 1 h at 37 °C. All solutions used were prepared in MilliQ water and the pH was adjusted to 4.5. After mixing, the pH was maintained constant at 7.4 by addition of ammonium hydroxide 37% solution. The precipitate was aged for 24 hours, while checking the pH of supernatant. The solid obtained was extracted by centrifugation, washed 3 times with Milli-Q water, and dried for 3 days at 37 °C. The obtained white powder was ground in a mortar and sieved (75 μm).

**X-ray diffraction (XRD)**

XRD profiles were collected using a diffractometer equipped with a 18 kW rotating anode (with a copper target), an asymmetric Johansson Ge(111) crystal to isolate monochromatic Cu $K\alpha_1$ radiation ($\lambda$ = 1.54056 Å), a horizontal 2θ/θ goniometer and a Silicon strip D/teX Ultra detector. Measurements were performed at room temperature in Debye-Scherrer geometry, using Lindemann glass capillaries to hold the samples. To minimize the impact of potential preferred orientation, the capillary was rotated during the measurements, to promote random orientation of the individual crystallites.

**Scanning Electron Microscopy (SEM)**

The surface morphology of the powders was investigated with a field emission scanning electron microscope (FESEM) using a Thermo Scientific™ Helios™ 5 UC Dual Beam system equipped with a Through Lens Detector (TLD), polarized to acquire Secondary Electrons for high resolution imaging in immersion mode. The powders were sonicated for 15 minutes, dispersed on a copper tape, and excess was removed.

**Fourier-transform infrared (FT-IR) spectral analysis**

FTIR spectroscopy was used to define the functional groups of Hap and Hap-Car derivatives. The spectra were acquired using a Thermo Scientific Nicolet iS10 FT-IR Spectrometer. The samples were mixed with KBr and pressed into pellets. The spectra were recorded with a resolution of 4 $cm^{-1}$ in the range of 4000–400 $cm^{-1}$ using 32 scans.

**Carnosine loading**

The peptide loading on the Hap-Car derivatives was determined using a spectrofluorimetric method [1]. Fluorescence spectra were performed using a VICTOR® Nivo™ multimode microplate reader at an excitation wavelength of 340 nm and an emission wavelength of 450 nm. Samples were treated with a solution of HCl (0.1 M)/ EDTA (0.2 M) (10:90) to achieve complete dissolution, and finally the pH was adjusted to 7 by adding NaOH. In a 384-well plate, 33 μL of the sample or a standard solution was aliquoted into the well of a microtiter plate, where 10 μL of a solution of 13 mM OPA and 7 μL of 20 mM NAC were previously added. The plate was shaken for 30 seconds before each measurement. The fluorescence intensity was then recorded at regular intervals (1 minute) for 30 minutes. Fluorescence values were converted to carnosine concentrations using a calibration curve generated from different mixtures of Car dissolved in HCl (0.1 M) / EDTA (0.2 M) (10:90). The determinations were carried out in triplicate. The determination of Car loading values was repeated after incubation periods of 24 hours and 4, 7 and 15 days in phosphate buffer. The resulting solid was subjected to centrifugation, after which the above procedure was repeated to determine the remaining carnosine.

**Inductively Coupled Plasma – Optical Emission Spectroscopy and Mass Spectrometry**

The concentration of copper was quantified using a Thermo Scientific iCAP 7400 Duo ICP-OES, this instrument was operating in axial and radial (so called Dual) mode with a $N_2$ purged polychromator to reduce molecular interferences. Two analytical wavelengths (i.e. Cu 324.754 and Cu 327.396) were used to confirm the concentrations and ensure spectra free from interferences. The concentrations especially the lower ones were confirmed by analysis of the samples with a Thermo Scientific iCAP RQ ICP_MS operating in kinetic

energy discrimination (KED) mode to quantify Cu at m/z 63 and 65. The calibration standards at 0, 1, 10, 50 and 100 µg/L were made in metal free polypropylene centrifuge tubes by mass not volume to ensure low blanks and reproducibility and correct for pipetting errors. The instrument detection limits (IDL) were low enough to allow 5-fold sample dilution which further reduced matrix effects. The calibrations had an excellent linearity, and the diluent (a mix of Triton X-100 (0.1% v/v), ammonia (1% v/v) and EDTA (0.1% m/v) in ultrapure 18M ohm water (Elga Lab Water, High Wycombe, UK) was chosen to keep the proteins and metals stable in solution while maintaining low blank concentrations.

**Cell culture and treatments**

The immortalized human fetal osteoblast cell line hFOB1.19 (hFOB, CRL-11372™) was purchased from American Tissue Type Culture (ATCC). hFOB1.19 cells were grown in complete DMEM/Ham's F12 medium supplemented with 10% fetal bovine serum (FBS), 2 mM sodium pyruvate (Gibco), 50 IU/mL penicillin, and 50 µg/mL streptomycin. Cells were cultured in a humidified incubator supplemented with 5% $CO_2$ at 37 °C. For the hFOB1.19 treatment, the cells were plated in starvation medium (DMEM/Ham's F12 medium supplemented with 1% FBS, 2 mM sodium pyruvate, 50 IU/mL penicillin/streptomycin) and treated for 4 days with different concentrations of Hap, Hap-Car10:1, Hap-Car2:1, Hap-Car1:1. To investigate the role of $Cu^{2+}$ in osteoblast differentiation, the cells were simultaneously treated with of 2,9-Dimethyl-4,7-diphenyl-1,10-phenanthroline disulphonic acid (BCS) (50 µM), an extracellular chelator of $Cu^{2+}$.

**Cellular cytocompatibility and proliferation**

The effect of Hap and Hap-Car treatments on cell viability was tested at 60–70% of cell confluence by incubation with the compounds at two concentrations (0,05 and 0,1 mg/mL) in the starvation medium for 4 days (4d). The viable cells were quantified by the reaction with 3-(4,5-dimethylthiazol-2-yl)-2,5-diphenyltetrazolium bromide (MTT method), as previously described [2]. After 90 min, the reaction was stopped by adding DMSO, and absorbance was measured at 569 nm (Varioskan® Flash Spectral Scanning Multimode Reader, Waltham, MA, USA). Results were expressed as the percentage of viable cells over the concentration of each compound. The experiments were repeated at least 3 times in triplicate and results expressed as mean ± SD. The changes in cell proliferation were quantified in hFOB1.19 cells through a label-free approach using the IncuCyte SX1 (Serial Number: IC 60068) live cell imaging system. hFOB1.19 cells were incubated with the compounds described earlier, and cell proliferation was assessed at 4dd. The phase

contrast image was collected with 10× objective, and data were then analyzed using the Incucyte® Artificial Intelligence (AI) Confluence Analysis Workflow, software v2022A (www.sartorius.com/en/products/live-cell-imaging-analysis/live-cell-analysis-software/incucyte-base-software) (Table S1), which quantifies cell surface area coverage as confluence values. The results were expressed as the proliferation rate between the control and treatments.

**Table S1.** Analysis of cell confluence. Selected parameters used to set the base analysis with the "Artificial Intelligence (AI)" mask.

| Segmentation adjustment | AI confluence |
|---|---|
| Cleanup | - |
| Area | 1000,00 $\mu m^2$ |
| Image channels | Phase |
| Magnification | 10× |

**Alizarin Red Staining**

The extent of extracellular matrix mineralization, a late marker of osteogenic differentiation, was evaluated on day 4 of differentiation by Alizarin Red S staining. The cells cultured in the presence of Hap and Hap-Car10:1, Hap-Car2:1, Hap-Car1:1 in starvation medium with or without 50 µM BCS were fixed in 4% paraformaldehyde in PBS (Santa Cruz, sc-281692) for 15 min at RT and then washed with $dH_2O$. Then the fixed cells were incubated in 40 mM Alizarin Red S in $dH_2O$ (pH 4.1; Sigma-Aldrich, Merck, Germany) for 15 min at RT. The unbound dye was washed away by $dH_2O$ (5 times for 10 min). The cell morphology and calcium deposits were imaged by a Leica DMI 6000B epifluorescence inverted microscope with Adaptive Focus Control at 20x magnification.

To extract the dye, the samples were incubated in 20% methanol and 10% acetic acid in water overnight at RT with gentle shaking. The solution that was obtained was transferred to a 96 multiwell plate and the absorbance of the solution was measured at 450 nm by a plate reader (Varioskan® Flash Spectral Scanning Multimode Reader, Waltham, MA, USA). A solution without cells was used as a blank control.

**Alkaline Phosphatase activity (ALP) assay**

The osteogenic activity of the Hap and Hap-Car10:1, Hap-Car2:1, Hap-Car1:1 in promoting osteogenic differentiation of hFOB1.19 cells after 4 days of incubation was evaluated by ALP activity assay. hFOB1.19

cells were treated with 0.05mg/mL Hap and its carnosine derivatives with or without 50 µM BCS in phenol red free medium (DMEM/Ham's F12 medium supplemented with 1% FBS, 2 mM sodium pyruvate, 50 IU/mL penicillin/streptomycin) and the ALP activity was measured using an ALP assay kit (Colorimetric; Abcam, ab83369) according to the manufacturer's instructions. Briefly, 80 µL of the conditioned culture media of samples was added to 50 µL of 5 mM p-nitrophenyl phosphate solution as a phosphatase substrate for ALP and was incubated at room temperature for 1 h. A solution without cells was used as a blank control. The absorption of the solution was measured at 405 nm by a plate reader (Varioskan® Flash Spectral Scanning Multimode Reader, Waltham, MA, USA).

**Enzyme-Linked Immunosorbent Assay (ELISA)**

Medium samples were collected after 4 days of exposure to Hap (0,05 mg/mL) and its derivatives with carnosine in starvation medium in the presence or in the absence of 50 µM BCS; samples were then centrifuged (14,000× g, 10 min) and supernatants were transferred into clean microtubes and stored at −80 °C until analysis.

The amount of BDNF and BMP2 secreted in the cell culture medium after 4 days was quantified using the Human BDNF Simple Step ELISA kit (Abcam (MA, USA); ab212166) and Human BMP2 ELISA kit (Thermo Fisher Scientific, EHBMP2), according to the manufacturer's instructions.

The level of osteocalcin release was determined from the cell culture media samples using a direct ELISA assay. The level of VEGF release was analyzed in the cell culture medium as previously described[3]. Polyvinyl chloride (PVC) microtiter plates were coated overnight at 4 °C with medium samples diluted 2:1 with carbonate/bicarbonate buffer (pH 9.6). Then plates were washed twice with PBS, blocked by a blocking buffer (5% BSA in PBS) for 2 hrs at room temperature, washed with PBS and incubated overnight with 1 µg/mL of detection antibody (anti-Osteocalcin, ab133612 (Abcam (MA, USA)), or anti-VEGF, code: sc-7269 (Santa Cruz Biotechnology, Inc.) in PBS supplemented with 1% BSA, washed again, then incubated for 2 hrs with HRP-conjugated secondary antibody and washed with PBS. The result was detected by 3,3′,5,5′-tetramethylbenzidine (TMB) solution after incubation for 30 min. The reaction was stopped by a solution of $H_2SO_4$ (2 M).

The optical density for all ELISA assays was measured at 450 nm by a plate reader (Varioskan® Flash Spectral Scanning Multimode Reader, Waltham, MA, USA).

**Immunocytochemistry**

To study BMP2 expression after treatment with Hap and Hap-Car 10:1, Hap-Car 2:1, Hap-Car 1:1 derivatives, hFOB1.19 cells were seeded in 96 multiwells and were treated for 4 days. Immunocytochemistry analysis was carried out according to the method described in a previous paper[4]. Briefly, cells were fixed, permeabilized and blocked with 0.2% gelatine in Dulbecco's Phosphate-Buffered Saline. BMP2 was detected by incubating overnight cells with rabbit anti-BMP2 antibodies (Abcam, code ab214821, dilution 1:250). After DPBS washing, cells were exposed for 1 h at room temperature to the secondary antibody (Goat anti-Rabbit IgG (H+L) Cross-Adsorbed Secondary Antibody, Alexa Fluor™ 488 (A11008, dilution 1:500; Thermo Fisher). Hoechst33342 (Molecular Probes, 1 μg $\mu L^{-1}$) was used to stain nuclear DNA. Images were analyzed under a Leica DMI 6000B epifluorescence inverted microscope with Adaptive Focus Control at 40x magnification. Images were taken at random locations throughout the area of the well for all the samples. Fluorescence quantification of anti-BMP2 immunostaining was carried out by using ImageJ Software 1.53 g (http://imagej.nih.gov/ij; Java 1.8.0_112(64bit)). Sixty green (BMP2)/blue (Hoechst33342) values were taken from twenty nuclear ROI (region of interest)/microscopic field, in 3 fields/well.

**Protein lysate preparation and Immunoblotting**

To analyze the expression of the osteogenic marker BMP2 and expression of the main copper homeostasis players (Ctr1, CCS, ATP7B) after Hap and Hap-Car10:1, Hap-Car2:1, Hap-Car1:1 treatment, hFOB1.19 cells were treated for 4 days or 48h, respectively. Cells incubation, sample preparation and western blot analysis were carried out according to the method described in previous papers [5]. Briefly, hFOB1.19 cells were collected, cell pellets were lysed and the protein concentrations in the supernatant were determined by Bradford’s method (Protein Assay Dye Reagent Concentrate, BioRad, Hercules, CA, USA). Proteins were separated by 4-20% Tris-Glycine gels (Bio-Rad, CA, USA) and transferred onto nitrocellulose membranes. Proteins were detected with specific primary antibodies by incubation overnight at 4°C (Table S2).

The appropriate infrared-dye labelled secondary antibodies were used to detect primary antibodies. The secondary goat anti-rabbit (Cat# 925-32211) and goat anti-mouse (Cat# 926-68070) labeled with IRDye 800 (1: 20000) and IRDye 680 (1:20000), respectively, were from LI-COR (Lincoln, NE, USA). The Odyssey Infrared Imaging System (LI-COR Biosciences, Lincoln, NE, USA) was used to scan the blot; quantitative densitometric analysis was performed by using ImageJ Software 1.53 g (http://imagej.nih.gov/ij; Java

1.8.0_112 (64bit)). The results were expressed as arbitrary densitometric units (A.D.U.) and the values were normalized to Actin or GAPDH expression levels, and presented as % of untreated cells control.

**Table S2.** Antibodies used for protein detection

| Antibody | Code | Company | Dilution |
|---|---|---|---|
| BMP2 | ab214821 | Abcam (MA, USA) | 1:3000 |
| Gapdh | ab8245 | Abcam (MA, USA) | 1:3000 |
| Ctr1 | ab129067 | Abcam (MA, USA) | 1:3000 |
| CCS | sc-55561 | SANTA CRUZ BIOTECHNOLOGY, INC. | 1:500 |
| ATP7B | H00000540 | Novus Biologicals | 1:500 |
| Actin | 4970 | Cell Signaling Technology (Danvers, MA, USA) | 1:2000 |

**Long term maintenance of Hap and Hap-Car in culture medium**

Hap, Hap-Car10:1, Hap-Car2:1, Hap-Car1:1 (0.05 mg/mL) were incubated in cell culture starvation medium for 2 weeks in a humidified incubator at 37°C in the absence of the hFob1.19 cells. After that Hap, Hap-Car 10:1, Hap-Car 2:1, and Hap-Car 1:1 were centrifuged, transferred into clean eppendorf tubes with PBS, centrifuged again and then added to hFob1.19 cells in a fresh starvation medium for 4 days. The effect of these Hap and all HapCar derivatives were investigated, evaluating again their cytocompatibility by MTT assay and BMP2 expression level by immunoblotting.

**Data and Statistical Analysis**

Results were expressed as the mean ± standard deviation of at least three experiments performed in triplicates. One-way analysis of variance (ANOVA) followed by Tukey's post-hoc test was used for comparisons between all groups, whereas the Student's t test was used to compare the means between two groups with or without BCS. All statistical analyses were performed with GraphPad Prism 6.0 software (GraphPad Software Inc., La Jolla, CA, USA). Differences were considered to be significant at p values $< 0.05$.

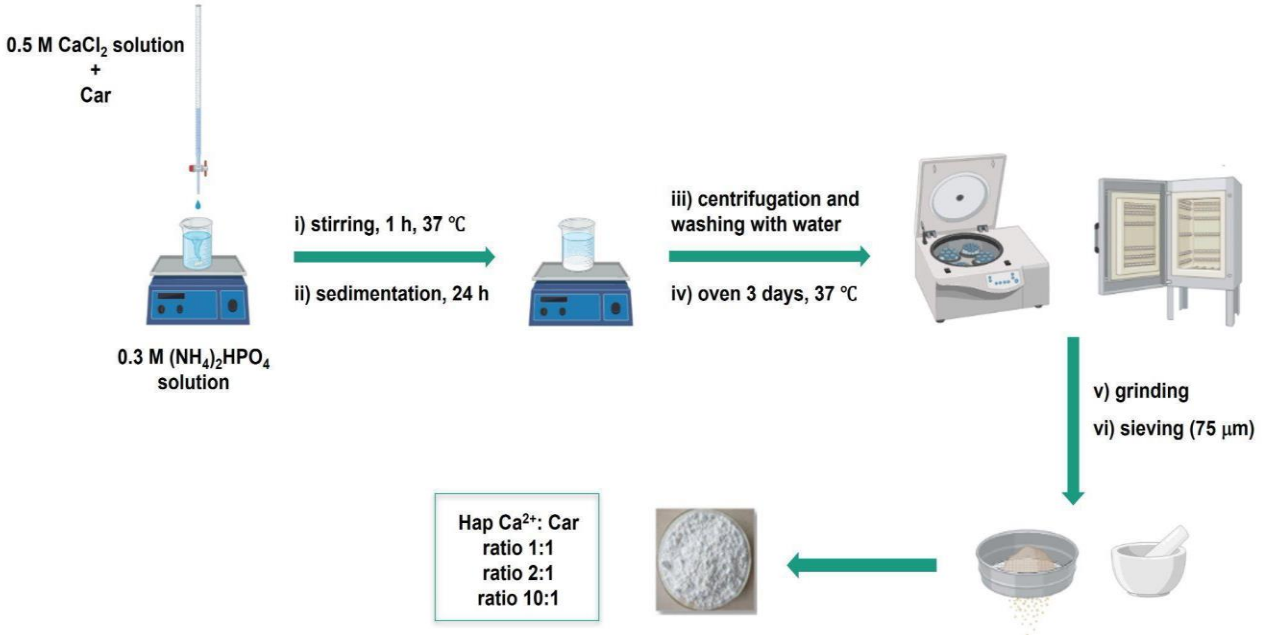


**Scheme 1.** Schematic representation of Hap-Car derivatives synthesis by co-precipitation.

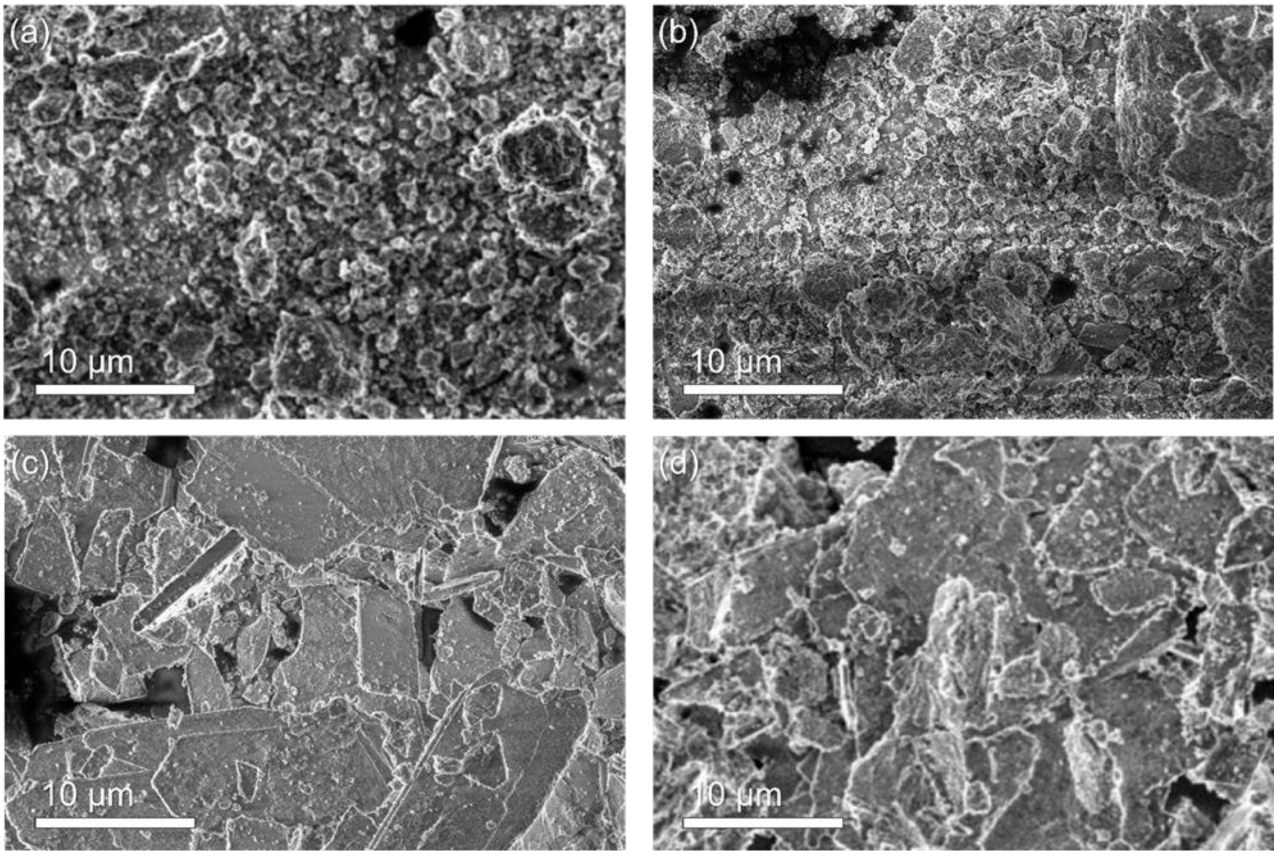


**Figure S1.** SEM comparison between Hap-Car derivatives at low magnification: (a) Hap-Car1:1, (b) Hap-Car2:1, (c) Hap-Car10:1, (d) Hap.

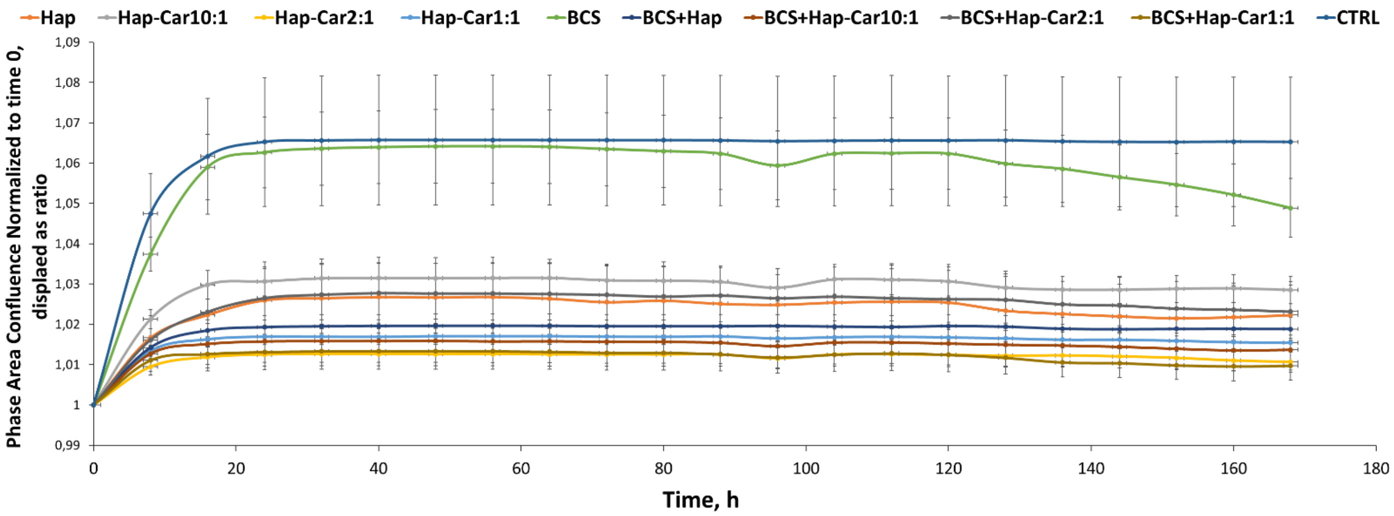


**Figure S2**. hFOB1.19 cell proliferation curves. Cells were treated with Hap, Hap-Car10:1, Hap-Car2:1 and Hap-Car1:1 in the presence or absence of BCS (50 µM). Cell proliferation was analyzed with the IncuCyte instrument. The phase area confluence was detected during 7d and calculated as total occupied area of cells vs time 0 by Incucyte Base Analysis with "Artificial Intelligence" mask for cell detection.

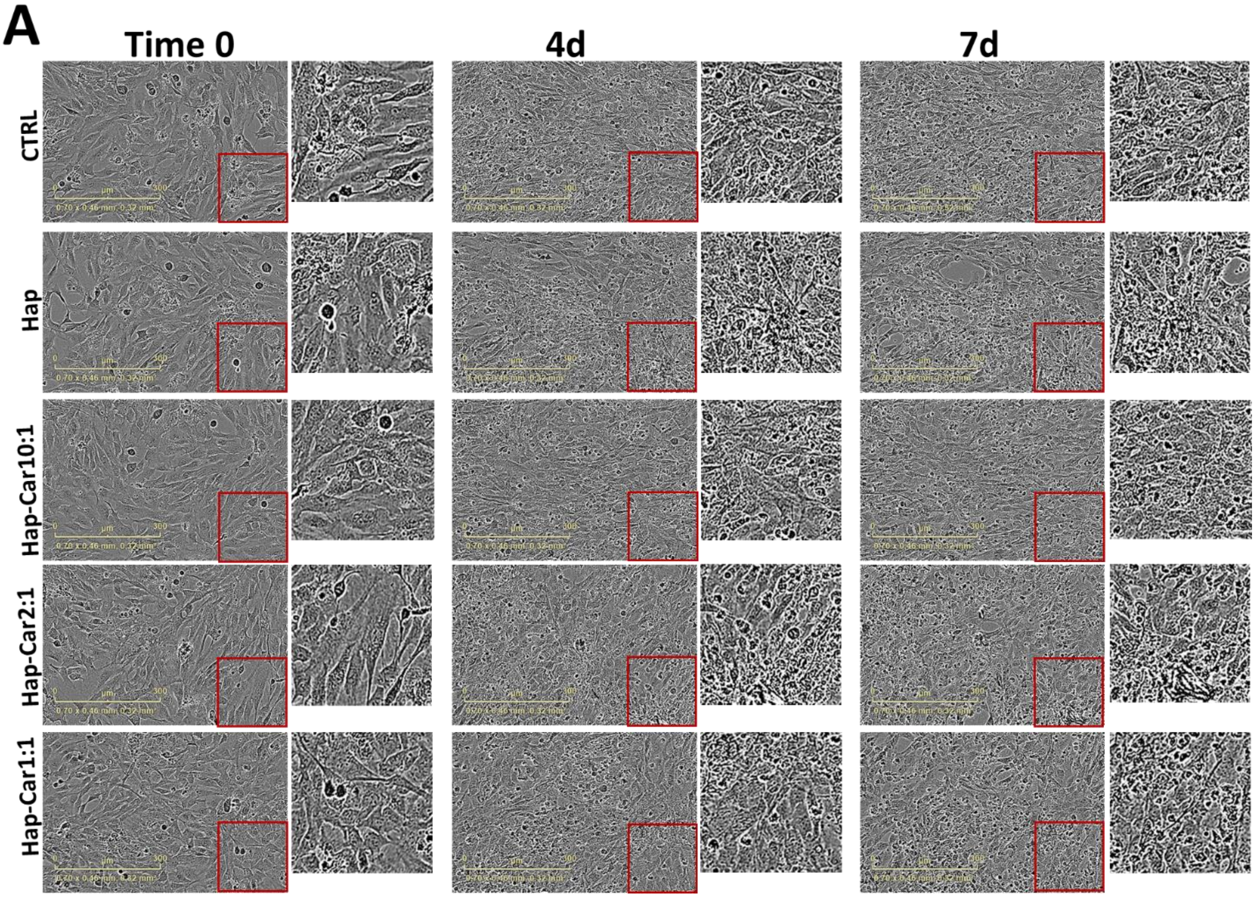
A
Time 0
4d
7d
CTRL
Hap
Hap-Car10:1
Hap-Car2:1
Hap-Car1:1

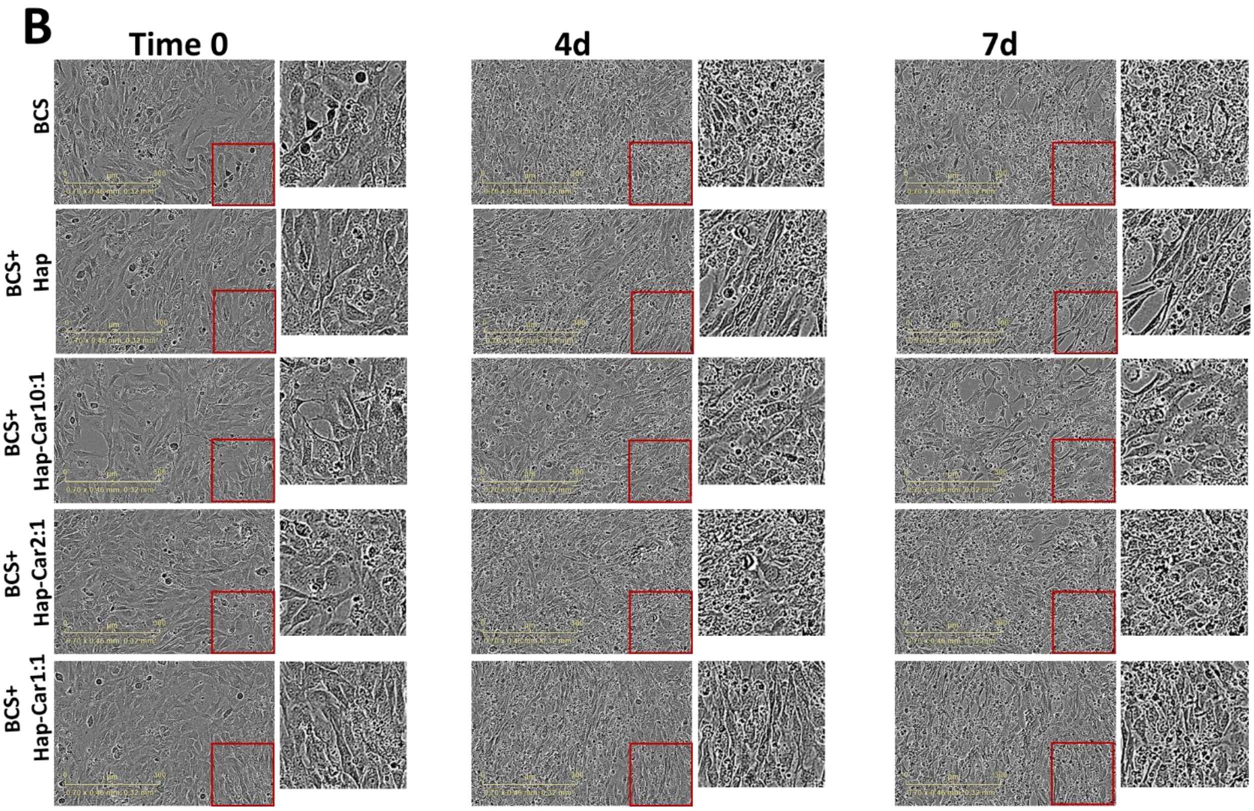


**Figure S3.** Representative images of hFOB1.19 cells morphology were captured using IncuCyte instrument. Cells were treated with Hap, Hap-Car10:1, Hap-Car2:1 and Hap-Car1:1 in the absence (A) or presence of BCS (B) (50 µM). Scale bar, 300 µm. Magnification 10x.

References

[1] F. Robert-Peillard, E. P. Barco, M. Ciulu, C. Demelas, F. Théraulaz, J.-L. Boudenne, B. Coulomb, *Microchemical Journal* **2017**, *133*, 216–221.

[2] I. Naletova, V. G. Nicoletti, D. Milardi, A. Pietropaolo, G. Grasso, *Metallomics* **2016**, *8*, 750–761.

[3] B. Tomasello, F. Bellia, I. Naletova, A. Magrì, G. Tabbì, F. Attanasio, M. F. Tomasello, W. R. L. Cairns, M. Fortino, A. Pietropaolo, V. Greco, D. La Mendola, S. Sciuto, G. Arena, E. Rizzarelli, *ACS Chemical Neuroscience* **2024**, *15*, 1755–1769.

[4] F. Bellia, V. Lanza, I. Naletova, B. Tomasello, V. Ciaffaglione, V. Greco, S. Sciuto, P. Amico, R. Inturri, S. Vaccaro, T. Campagna, F. Attanasio, G. Tabbì, E. Rizzarelli, *Antioxidants* **2023**, DOI 10.3390/antiox12081632.

[5] I. Naletova, G. I. Grasso, C. Satriano, A. Travaglia, D. La Mendola, G. Arena, E. Rizzarelli, *Metallomics* **2019**, *11*, 1567–1578.
